# The different tolerance to magnesium deficiency of two grapevine rootstocks relies on the ability to cope with oxidative stress

**DOI:** 10.1186/s12870-019-1726-x

**Published:** 2019-04-16

**Authors:** Sonia Livigni, Luigi Lucini, Davide Sega, Oriano Navacchi, Tiziana Pandolfini, Anita Zamboni, Zeno Varanini

**Affiliations:** 1 Biotechnology Department, University of Verona, Strada Le Grazie 15, 37134 Verona, Italy; 2 Department for Sustainable Food Process, Università Cattolica del Sacro Cuore, via Emilia Parmense 84, Piacenza, Italy; 3 Vitroplant Italia, via Loreto 170, 47521 Cesena, Italy

**Keywords:** Magnesium deficiency, Grapevine rootstocks, Metabolome and transcriptome

## Abstract

**Background:**

Magnesium (Mg) deficiency causes physiological and molecular responses, already dissected in several plant species. The study of these responses among genotypes showing a different tolerance to the Mg shortage can allow identifying the mechanisms underlying the resistance to this nutritional disorder. To this aim, we compared the physiological and molecular responses (e.g. changes in root metabolome and transcriptome) of two grapevine rootstocks exhibiting, in field, different behaviors with respect to Mg shortage (1103P, tolerant and SO4 susceptible).

**Results:**

The two grapevine rootstocks confirmed, in a controlled growing system, their behavior in relation to the tolerance to Mg deficiency. Differences in metabolite and transcriptional profiles between the roots of the two genotypes were mainly linked to antioxidative compounds and the cell wall constituents. In addition, differences in secondary metabolism, in term of both metabolites (e.g. alkaloids, terpenoids and phenylpropanoids) and transcripts, assessed between 1103P and SO4 suggest a different behavior in relation to stress responses particularly at early stages of Mg deficiency.

**Conclusions:**

Our results suggested that the higher ability of 1103P to tolerate Mg shortage is mainly linked to its capability of coping, faster and more efficiently, with the oxidative stress condition caused by the nutritional disorder.

**Electronic supplementary material:**

The online version of this article (10.1186/s12870-019-1726-x) contains supplementary material, which is available to authorized users.

## Background

Magnesium (Mg) is an essential macronutrient playing a key role in plant growth and development. It is the most abundant free divalent cation in the cytosol, and it is involved into chlorophyll biosynthesis [1]; Mg is also an essential cofactor for the activity of several enzymes, as for instance RNA polymerases, ATPases, protein kinases, phosphatases, glutathione synthase and carboxylases [2–6]. In addition, Mg is important for protein synthesis being the bridging element for ribosome aggregation [7] and it also represents a regulator of stromal pH [8, 9].

Magnesium concentration in soil solution generally ranges between 0.5 and 8.0 mM [10], depending on soil characteristics and environmental or anthropogenic factors [11]. There are two main reasons causing Mg deficiency: absolute deficiency and cation competition [11]. All these conditions result in a lower accumulation of Mg in plant tissues with a consequent reduction of crop productivity and quality [12].

The physiological effects of Mg deficiency were well described in different species revealing an impact on growth parameters [13–18], sugar accumulation in source leaves [19–21] and antioxidant system [22–28]; however, the responses generally depend on the plant species, age and the system used to induce Mg deficiency.

Plant responses to Mg deficiency have been also analyzed in terms of changes in transcriptome [29–32], proteome [33, 34] and metabolome [35] in root and/or shoot tissues. In addition, miRNAs putatively involved into the responses to Mg deficiency were identified in *Citrus* leaf and root tissues [36, 37]. As far as root tissues are concerned it has been ascertained that Mg deficiency affected transcripts, proteins and metabolites involved into oxidative stress. In fact, among the long-term responses to Mg shortage in roots of *Arabidopsis thaliana* was observed an increase of the dehydroascorbate:ascorbate ratio and an upregulation of genes encoding thioredoxin (TRX), glutaredoxin (GRX) and glutathione-S-transferase (GST) [30]. Modulation of proteins and transcripts involved into the same response was also recorded in roots of *Citrus sinensis* [33] and *Citrus reticulata* [32], respectively. The shortage of Mg caused also an impairment of other processes such as respiration and metabolism of carbohydrates and energy as suggested by the modulation of hexokinase (HK), pyruvate decarboxylase (PDC), phosphoglycerate kinase (PGK), pyruvate dehydrogenase (PDH) and ATP synthase in *Citrus sinensis* [33]. In addition, a strong upregulation of a transcripts encoding an ATP synthase subunit β was observed in response to Mg deficiency also in *Citrus reticulata* [32]. Furthermore, Mg deficiency caused a decrease of organic acids as pyruvic, citric, 2-keto-glutaric, succinic, fumaric and malic in soybean roots [35]. The cell wall and cytoskeleton have been recorded as other targets of Mg shortage. It was in fact shown that changes in the abundance of proteins (e.g. actin 1, villin 3 and tubulin γ-1 chain) [33] and transcripts (cell wall protein-like, α-(1,4)-fucosyltransferase and endo-1,4-β-D-glucanase) [32] occurred in response to this nutritional disorder in *Citrus*.

Notwithstanding, to the best of our knowledge, the molecular events taking place during Mg starvation in genotypes differing in their tolerance to Mg shortage have not been described so far. The results of similar experiments could help dissecting the processes underlying the different tolerance to Mg deficiency, considering that, at present, the molecular bases accounting for these differences are still lacking. To this aim, we compared the physiological and molecular responses (e.g. changes in metabolome and transcriptome) of two grapevine rootstocks, Paulsen 1103 (1103P) and Selection Oppenheim 4 (SO4), exhibiting different tolerance to Mg deficiency, being tolerant and susceptible, respectively [38]. This kind of approach is particularly relevant to viticulture as grapevine is one of the world’s most economically important and cultivated fruit crop. It is in fact well recognized that Mg-deficiency in grapevine is a widespread nutritional disorder causing serious loss of production [39].

## Results

### Physiological responses to Mg deficiency of 1103P and SO4

**Fig. 1:**
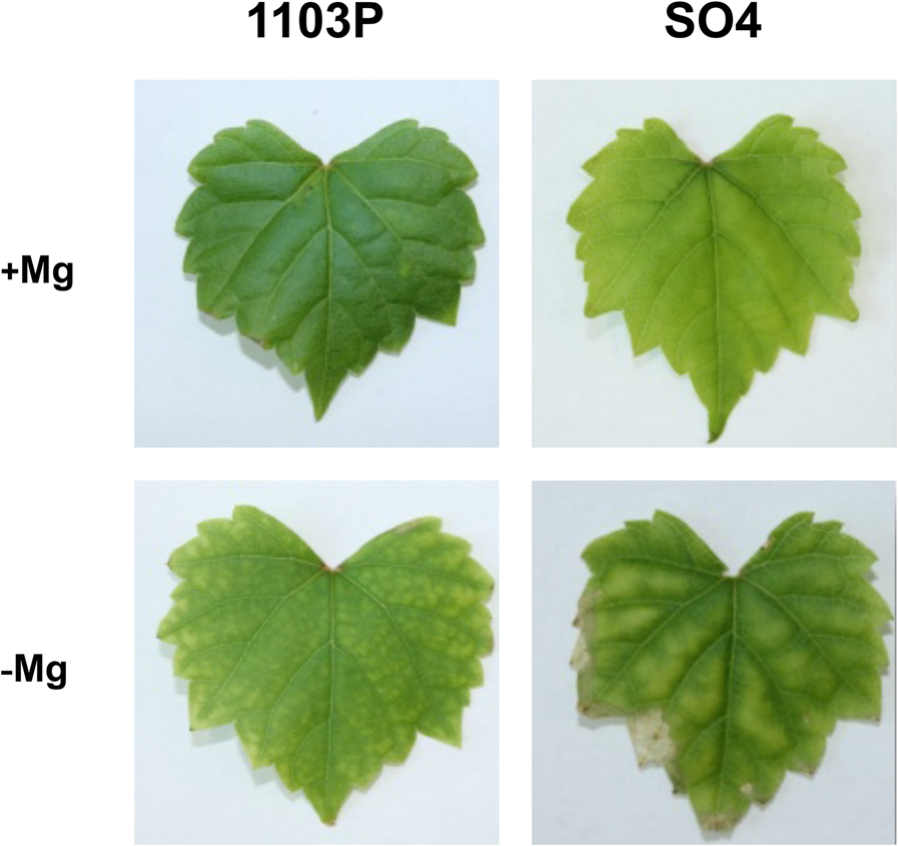
Details of old leaf of SO4 and 1103P cuttings grown for 14 days in presence (+Mg) and absence of Mg (−Mg)

**Fig. 2:**
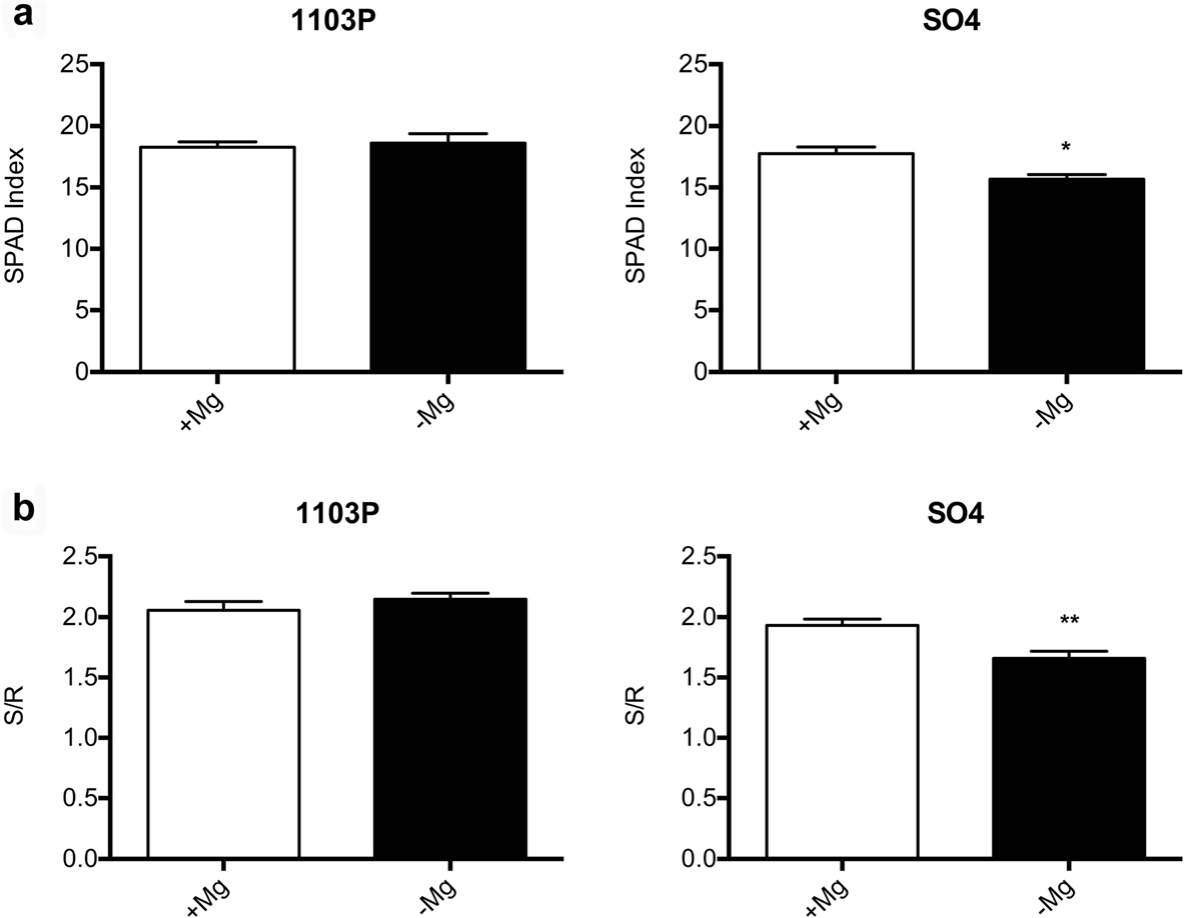
Mean SPAD index (**a**) and S/R (**b**) values of SO4 and 1103P microcuttings grown for 14 days in presence (+Mg) and absence of Mg (−Mg). Data were expressed as mean ± SEM of data from three growth independent experiments (*n* = 3). The statistical significance was determined by means of Student’s t-test. (∗*P* < 0.05) using the GraphPad InStat Program (version 5.0)

**Fig. 3:**
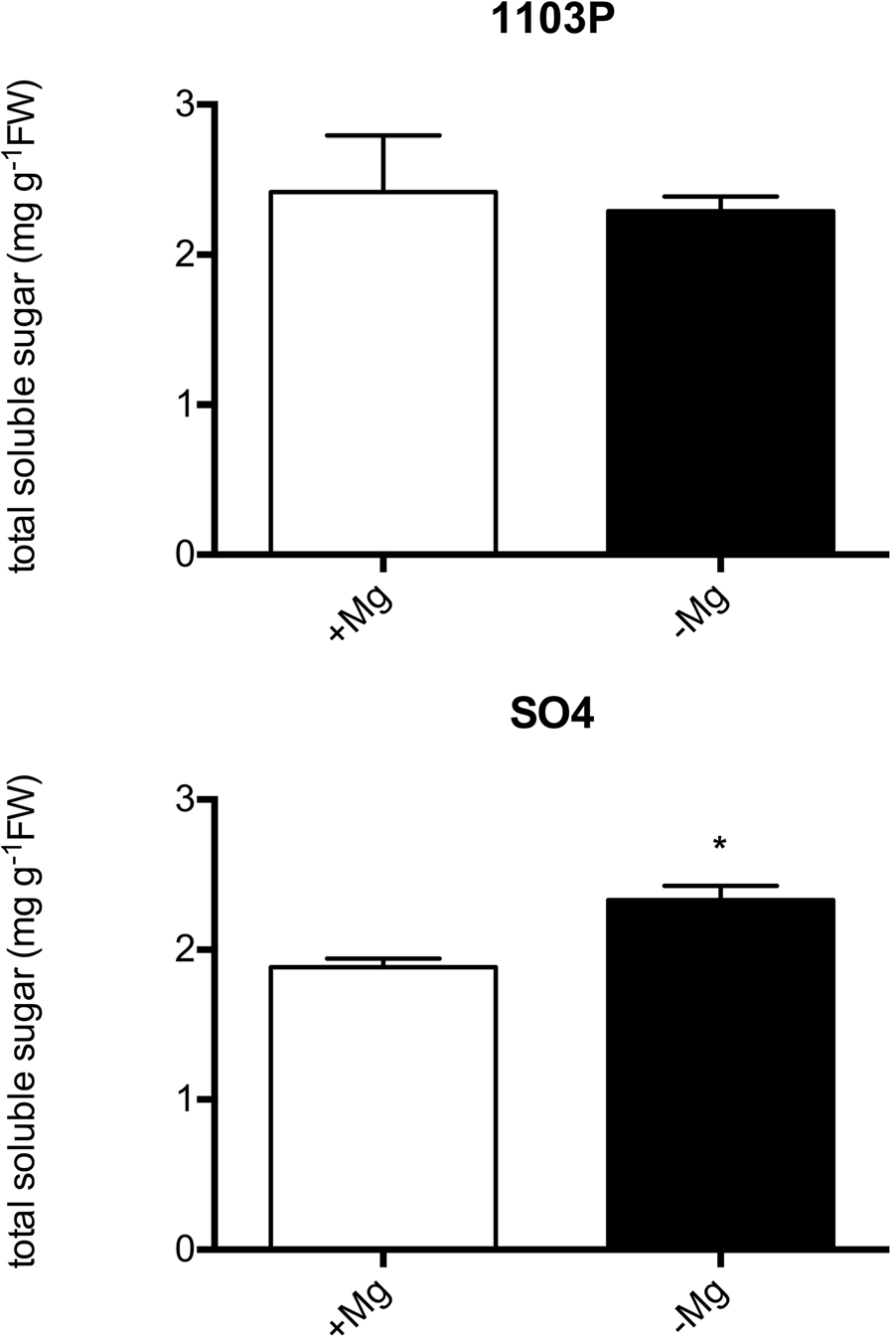
Total shoot soluble sugar concentration of SO4 and 1103P microcuttings grown for 14 days in presence (+Mg) and absence of Mg (−Mg). Data were expressed as mean ± SEM of data from three growth independent experiments (*n* = 3). The statistical significance was determined by means of Student’s t-test. (∗*P* < 0.05) using the GraphPad InStat Program (version 5.0)

The physiological responses to Mg shortage of the two grapevine rootstocks, SO4 and 1103P, reported as susceptible and tolerant to Mg paucity respectively, were evaluated using microcuttings grown in hydroponics. The experiment was carried out for 14 days by cultivating the plants with or without Mg. After this time, the typical symptoms of Mg deficiency (e.g. the interveinal chlorosis in old leaves) were visible in the leaves of both rootstocks, grown in the absence of Mg (Fig. 1). However, Mg deficiency symptoms were more severe in old leaves of SO4 that showed necrotic spots near the margin of the basal leaves (Fig. 1). This observation was confirmed by the significant decrease in SPAD index values of SO4 plants grown in the absence of Mg (Fig. 2a). In addition, the 14-day Mg shortage period caused a decrease of S/R ratio (Fig. 2b) and a significant increase in shoot sugar accumulation only in the susceptible SO4 rootstock (Fig. 3).

**Fig. 4:**
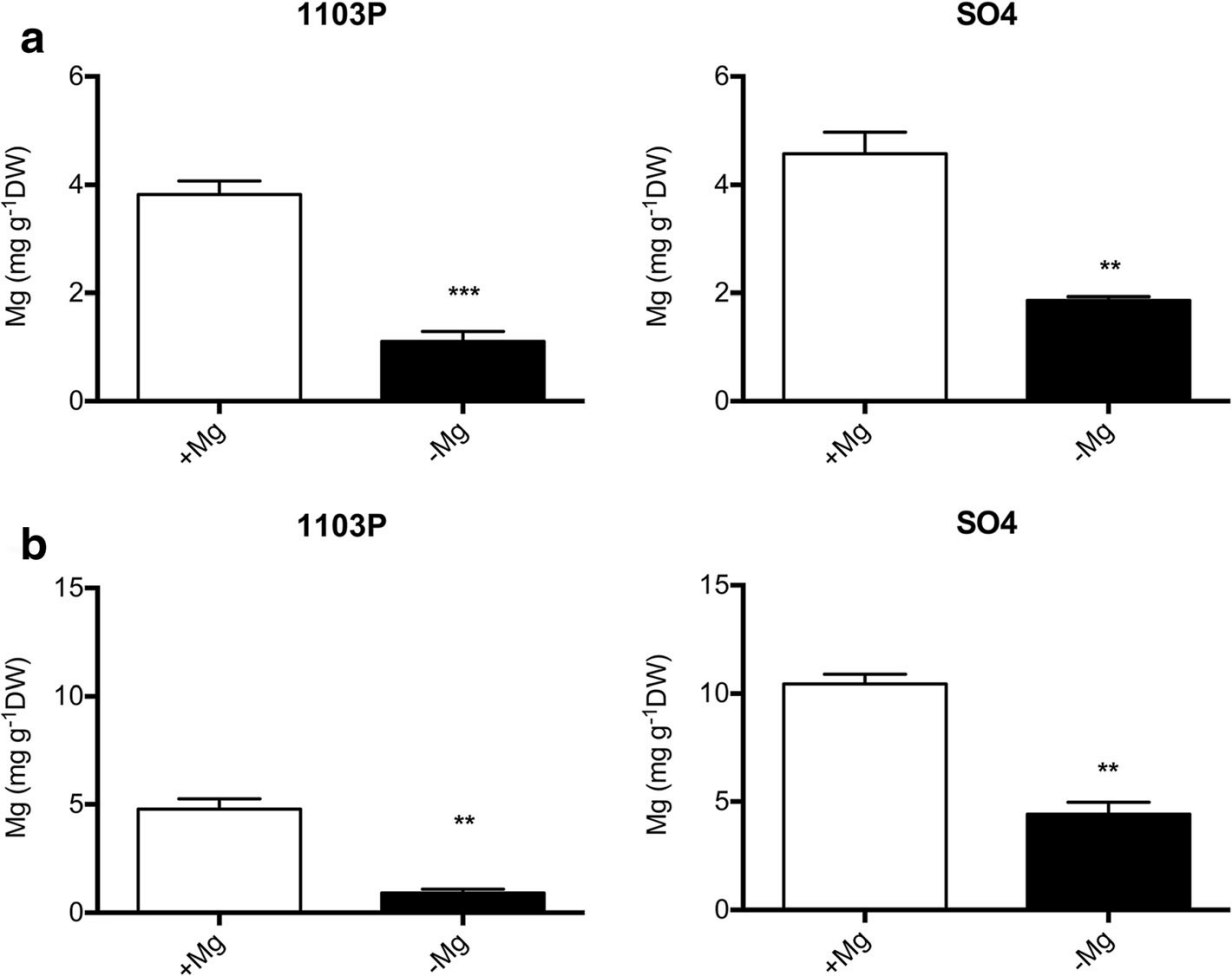
Shoot (**a**) and root (**b**) Mg concentration of SO4 and 1103P microcuttings grown for 14 days in presence (+Mg) and absence of Mg (−Mg). Data were expressed as mean ± SEM of data from three growth independent experiments (*n* = 3). The statistical significance was determined by means of Student’s t-test. (∗∗*P* < 0.01, ∗∗∗*P* < 0.001) using the GraphPad InStat Program (version 5.0)

Tissue concentrations of Mg are reported in Fig. 4; a statistically significant decrease in Mg concentration in shoots of both SO4 and 1103P was recorded when plants were grown under macronutrient shortage (Fig. 4a). Similar behavior was observed for the root Mg concentration (Fig. 4b). Interestingly, root Mg levels were higher in SO4 than in 1103P in both nutritional conditions (Fig. 4b).

### Metabolic responses to Mg deficiency in roots of SO4 and 1103P

**Fig. 5:**
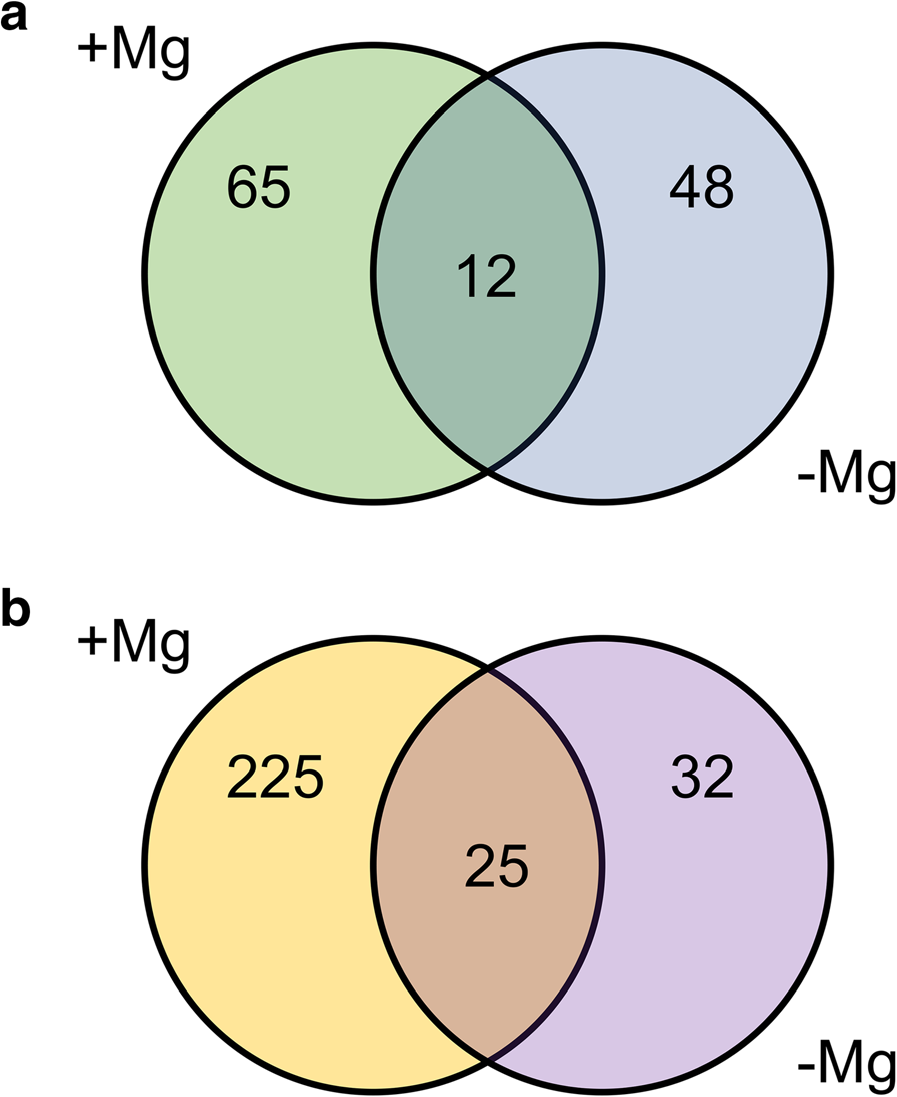
Metabolites differentially abundant in roots between 1103P and SO4 depending on the growth condition. (**a**) Metabolites specifically modulated at 4 days in presence (+Mg) and absence (−Mg) of Mg and modulated between the two genotypes in both nutritional conditions. (**b**) Metabolites specifically modulated at 14 days in presence (+Mg) and absence (−Mg) of Mg and modulated between the two genotypes in both nutritional conditions

**Fig. 6:**
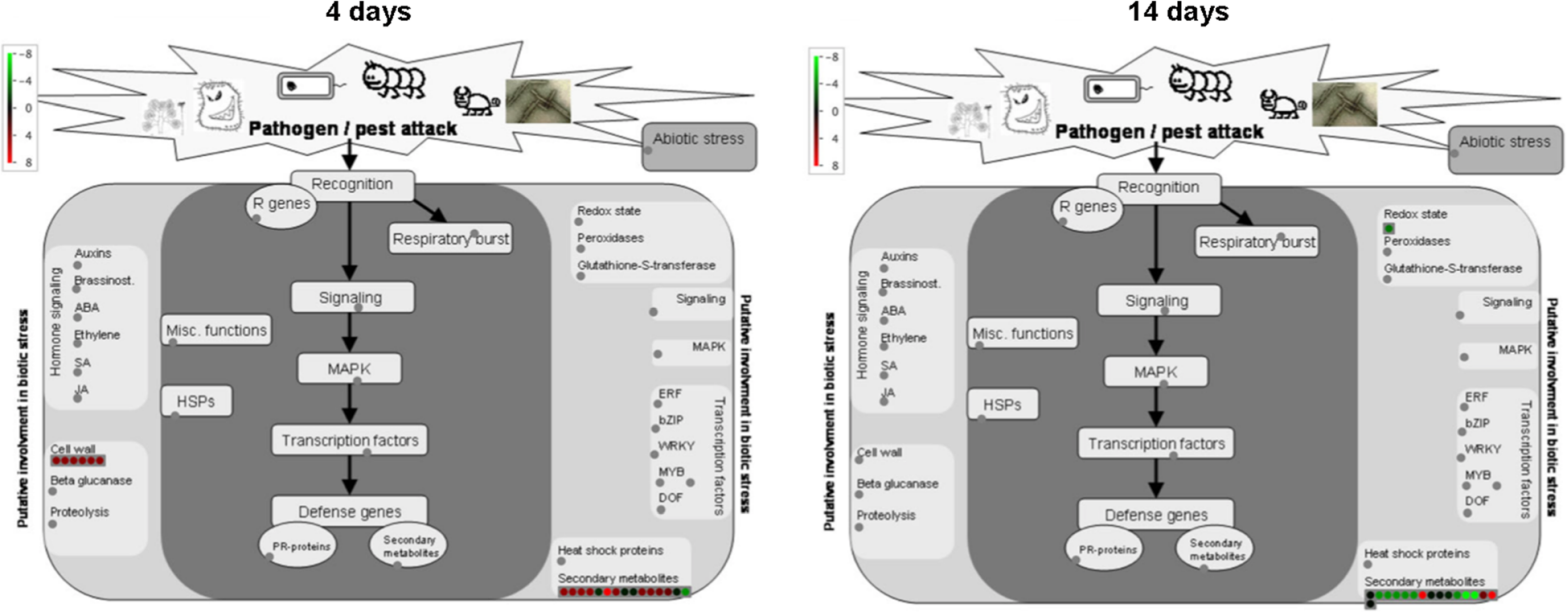
Differentially abundant metabolites between 1103P and SO4 linked to stress responses. The Log_10_(ratio) is shown by the color scale (green indicates a decrease and red an increase in metabolite abundance between 1103P and SO4). The analysis was carried out using MapMan software (https://mapman.gabipd.org/)

Changes in metabolomic profiles in roots of both grapevine rootstocks were evaluated in relation to the two nutritional conditions (+Mg and -Mg) through GC/MS and UHPLC-ESI/QTOF-MS for primary and secondary metabolites, respectively. The whole list of differentially accumulated metabolites, as selected by Volcano Plot analysis, is provided as supplementary information (Additional file 1: Table S1). The metabolic profiles of 1103P and SO4 microcuttings grown in +Mg, differed for the abundance of 77 and 250 metabolites after 4 and 14 days, respectively (Table 1, Additional file 2: Table S2 and Additional file 3: Table S3). Considering -Mg condition, the two genotypes differed at the two sampling time-points for 60 and 57 compounds, respectively (Table 1, Additional file 2: Tables S2 and Additional file 3: Table S3). By the means of Venn diagram, we identified the differentially abundant metabolites that distinguish the two rootstocks under the different nutritional conditions (+Mg, −Mg) and those that depends on the genetic background only (Fig. 5). In particular, we focused on the 48 and 32 metabolites that were differentially abundant at 4 (Fig. 5a) and 14 days (Fig. 5b) only in the roots of plants grown in -Mg conditions. At 4 days after treatments, 34 metabolites out of 48 were more abundant in 1103P as compared to SO4, whilst 14 were less abundant; at 14 days after treatments, 12 metabolites out of 32 were more abundant in 1103P with respect to SO4, whereas 20 were less represented (Additional file 4: Table S4). Considering the distribution in the main chemical classes, we observed that carbohydrate (41.18%) and terpenoids (11.76%) were the most represented classes for the “more abundant metabolites” in 1103P identified at 4 days. On the other hand, amino acids or their derivatives (28.57%), carbohydrates (14.29%), gibberellin (14.29%) and phenylpropanoids (14.29%) were the most represented classes for the “less abundant metabolites” (Additional file 4: Figure S1). After 14 days, coenzyme A-activated compounds (16.67%), glucosinolates (16.67%) and lipids (16.67%) were the most abundant classes for the metabolites showing higher levels in 1103P relative to SO4, whilst terpenoids (30%), alkaloids (20%) and phenylpropanoids (20%) were the most represented classes considering the “less abundant metabolites” in 1103P (Additional file 4: Figure S2). A selection of these metabolites is reported in Table 2. Considering all the differentially abundant metabolites under Mg deficiency in relation to the stress responses (Fig. 6), a higher level of metabolites belonging to secondary metabolism in the roots of the tolerant 1103P genotype at 4 days was observed. On the other hand, the production of secondary metabolites was delayed in the susceptible rootstock as supported by the major content of secondary compounds recorded in SO4 at 14 days (Additional file 4: Figure S3). In particular, the roots of 1103P produced more alkaloid-like compounds after 4 days, whilst the levels of this class of compounds and terpenoids were reduced at 14 days (Additional file 4: Figure S3). Furthermore, glucosinolates were specifically accumulated in the tolerant genotype 1103P at 14 days (Additional file 4: Figure S3). Differences in the responses to stress between the two rootstocks were also highlighted by the higher production of compounds involved into cell wall metabolism that were more abundant in 1103P after 4 days of Mg deficiency (Fig. 6).

**Table 1:** Number of more and less abundant metabolites between 1103P and SO4 at 4 and 14 days

	1103P + Mg vs SO4 + Mg	1103P -Mg vs SO4 -Mg
**4 days**		
More abundant	40	41
Less abundant	37	19
**14 days**		
More abundant	85	25
Less abundant	165	32

**Table 2:** Selection of metabolites differentially accumulated in roots between 1103P and SO4 at 4 and 14 days of growth under Mg deficiency

Metabolite	FC
**1103P vs SO4 at 4 days**	
*Alkaloids*	
(S)-corytuberine	10,080.12
1,2-dehydroreticulinium	10,080.12
Norephedrine	5055.84
*Amino acids or their derivatives*	
Homomethionine	−7.34
L-DOPA	−32,643.51
* Carbohydrates*	
α-D-xylose	23,691.90
β-D-xylose	23,691.90
α-L-arabinofuranose	23,691.90
β-L-arabinopyranose	23,691.90
Maltohexaose	− 287,939.56
* Cofactors*	
Molybdenum cofactor	7.38
* Gibberellins*	
Gibberellin A12	–22.72
Methyl gibberellin A9	–22.72
* Nucleic acid components*	
ppGpp	−131.55
*Phenylpropanoids*	
Ferulate	18,227.24
Leachianone G	−7.82
Pelargonidin 3-O-(6-O-malyl)-β-D-glucoside	16.00^a^
*Terpenoids*	
1′-hydroxy-γ-carotene	41,946.68
β-cryptoxanthin	41,946.68
ε-carotene-3-diol	41,946.68
Kauralexin B2	−22.72
β-carotene 15,15′ epoxide	41,946.68
*Vitamins*	
Thiamin	−8.64
**1103P vs SO4 at 14 days**	
*Alkaloids*	
(R)-N-methylcoclaurine	−28,523.96
(S)-N-methylcoclaurine	−28,523.96
S-cheilanthifoline	−16.00^a^
Senecionine	− 16.00
* Amino acids or their derivatives*	
Allylcysteine	− 4892.33
*Aromatic compounds*	
L-arogenate	−2.51
*Lipids*	
1–18:1–2-18:3-phosphatidylcholine	46,713,708.00
*Terpenoids*	
Loganin	−2.49
(−)-menthol	−39,919.66
(+)-isomenthol	−39,919.66
(+)-neoisomenthol	−39,919.66
(+)-neomenthol	−39,919.66
(S)-(−)-citronellol	−39,919.64
4,9,13-trimethyltetradeca-2,4,6,8,10,12-hexaene-1,14-dial	8299.46
* Phenylpropanoids*	
3,5-dihydroxyanisole	−4.45
4-hydroxycinnamic acid	−16.00
Feruloyl-CoA	16.00
Isoliquiritigenin 4′-glucoside	−3.84
p-coumaroyltyramine	−11.51

^a^According to Mass Profiler Professional output, FC = 16.00 and FC = − 16.00 denote very high and a very low FC (fold change), respectively

### Transcriptional responses to Mg deficiency in roots of SO4 and 1103P

**Fig. 7:**
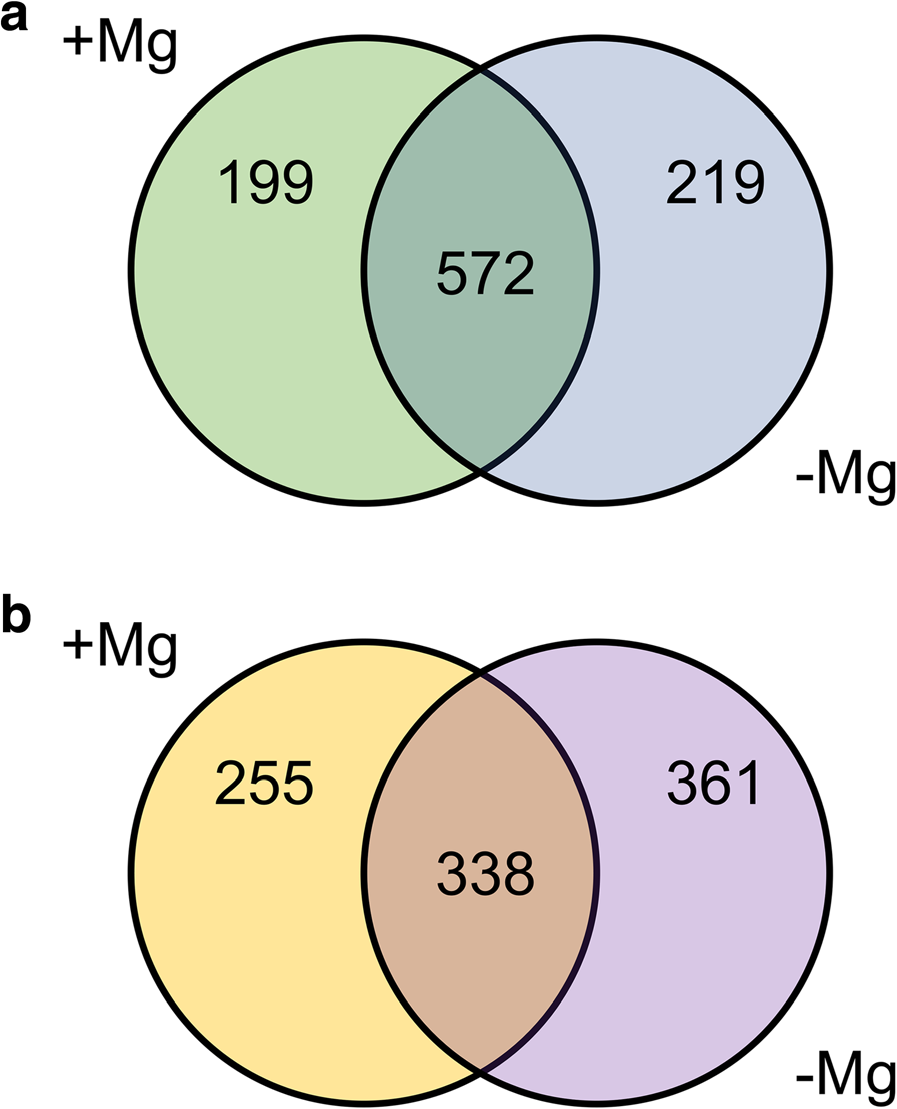
Transcripts differentially expressed in roots between 1103P and SO4 depending on the growth condition. (**a**) Transcripts specifically modulated at 4 days in presence (+Mg) and absence (−Mg) of Mg and modulated between the two genotypes in both nutritional conditions. (**b**) Transcripts specifically modulated at 14 days in presence (+Mg) and absence (−Mg) of Mg and modulated between the two genotypes in both nutritional conditions

**Fig. 8:**
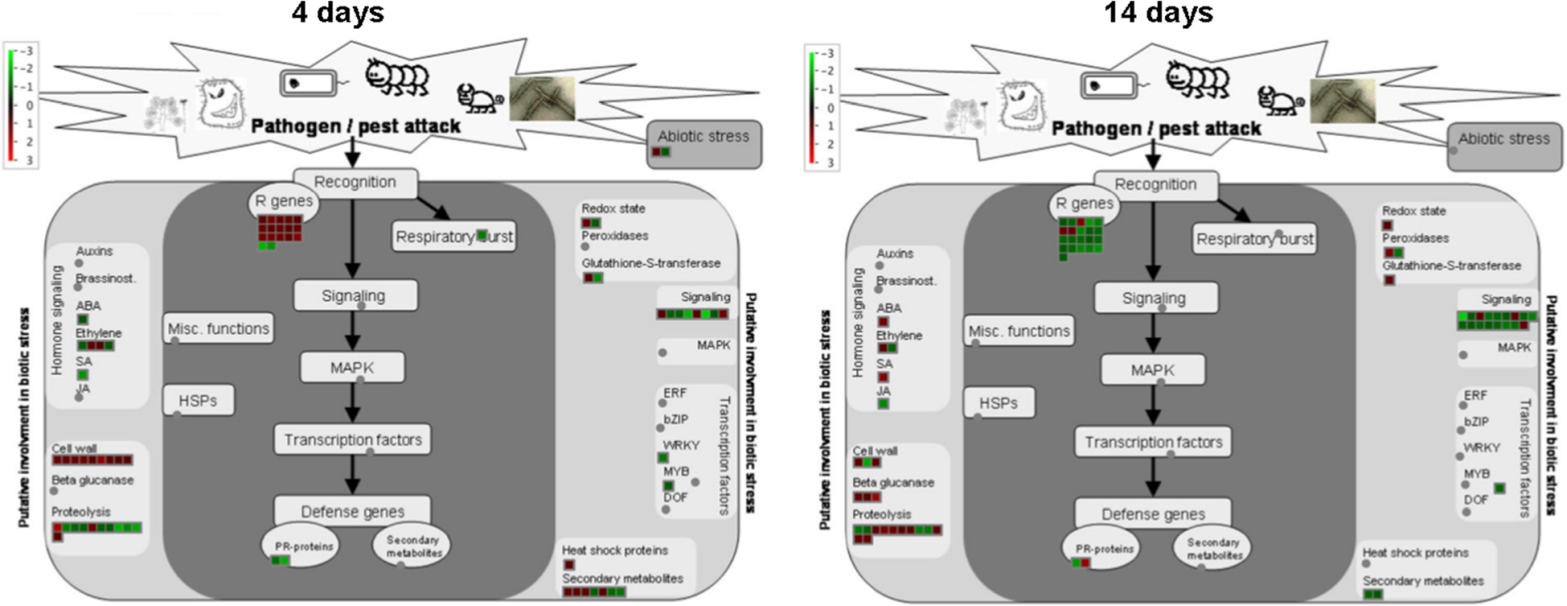
Differentially expressed transcripts between 1103P and SO4 linked to stress responses. The Log_2_(ratio) is shown by the color scale (green indicates a decrease and red an increase in transcript abundance between 1103P and SO4). The analysis was carried out using MapMan software (https://mapman.gabipd.org/)

The transcriptional differences between the two rootstocks in relation to Mg nutritional status were identified through microarray analysis. Different comparisons between transcriptional profiles were carried out using Linear Models for MicroArray [40] (adjusted *P*-value of 0.05 and a |Log_2_(T/C)| ≥ 1; Table 3 and Additional file 5: Table S5). In particular, 771 and 791 transcripts resulted differentially modulated in roots of 1103P and SO4 after 4 days of growth in presence and absence of Mg, respectively. The transcriptional differences between 1103P and SO4 after 14 days of growth in presence and absence of Mg, consisted in 593 and 699 transcripts. In addition, by comparing the lists of differentially expressed transcripts through Venn diagram (Fig. 7; Additional file 6: Table S6 and Additional file 7: Table S7), we pinpointed transcripts specifically modulated in presence or absence of Mg and commonly modulated under both nutritional conditions after 4 and 14 days. The attention was paid to transcripts modulated only under Mg shortage (219 after 4 days and 361 after 14 days). At 4 days, 115 and 104 transcripts resulted up- and down-regulated, respectively, when the two rootstocks were compared (Additional file 6: Table S6). Considering the second sampling time-point, we identified 128 positively and 233 negatively modulated transcripts between the two genotypes (Additional file 7: Table S7). A Singular Enrichment Analysis (SEA) was performed on transcripts encoding proteins involved in known biological processes using agriGO v2.0 [41]. This analysis showed that the more significant enriched GO terms for the up-regulated transcripts at 4 days were “secondary metabolic process” and “response to stress” (Additional file 8: Figure S4) whilst “cellular homeostasis” was the more enriched term for the down-regulated transcripts (Additional file 8: Figure S5). For the second sampling time-point (14 days), only “secondary metabolic process” resulted significantly enriched for the up-regulated set of transcripts (Additional file 8: Figure S6). Considering the down-regulated transcripts, “secondary metabolic process” and “responses to stimulus” were the highest significant enriched GO terms (Additional file 8: FigureS7). A selection of differentially expressed transcripts between the two rootstocks is reported in Table 4. A further insight into the data were obtained by analyzing the differentially expressed transcripts at both sampling time-points in relation to stress responses (Fig. 8). The tolerant genotype 1103P showed a higher engagement of transcripts involved into secondary metabolism, particularly evident at 4 days of treatment (Fig. 8 and Additional file 8: Figure S8). In addition, the faster response of this rootstock to the Mg shortage was also evident on the basis of a positive modulation of transcripts involved into cell wall metabolism at 4 days as previously observed for the metabolite dataset (Figs. 6, and 8). Furthermore, in the tolerant genotype Mg shortage positively affected R genes at 4 days whilst this response was delayed in the susceptible SO4 (Fig. 8). Conversely, transcripts playing a role in signaling were mostly up-regulated in SO4 roots particularly at 14 days (Fig. 8).

**Table 3:** Number of up- and down-regulated transcripts between 1103P and SO4 at 4 and 14 days

	1103P + Mg vs SO4 + Mg	1103P -Mg vs SO4 -Mg
**4 days**		
Up-regulated	412	421
Down-regulated	359	370
**14 days**		
Up-regulated	183	247
Down-regulated	410	452

**Table 4:** Selection of transcripts differentially expressed in roots between 1103P and SO4 at 4 and 14 days of growth under Mg deficiency

V1 Genome ID	Functional annotation	Log_2_(ratio)
**1103P vs SO4 at 4 days**		
*Cell wall organization and biogenesis*		
VIT_00s0414g00010	cellulose synthase CSLE1	1.19
VIT_00s0469g00040	cellulose synthase CSLE1	1.30
VIT_00s0531g00060	cellulose synthase CSLE1	1.01
VIT_00s1213g00010	cellulose synthase CSLE1	1.23
VIT_00s1213g00020	cellulose synthase CSLE1	1.19
VIT_01s0127g00850	polygalacturonase BURP	1.02
VIT_04s0044g01010	pectinesterase family	1.03
VIT_05s0020g02540	endo-1,3;1,4-beta-D-glucanase precursor	1.11
VIT_10s0116g00590	pectinesterase family	1.04
VIT_12s0059g00960	cellulose synthase CSLB04	1.71
VIT_12s0059g00990	cellulose synthase CSLB04	1.06
VIT_12s0059g00990	cellulose synthase CSLB04	1.09
*Cellular process*		
VIT_09s0002g06470	CYP81B2v1	−1.16
VIT_09s0002g06480	CYP81B2v1	−1.00
*Establishment of localization*		
VIT_08s0007g00030	cation/hydrogen exchanger 20 (CHX20)	−1.27
VIT_11s0103g00010	potassium-sodium symporter HKT2	1.23
*Homostatic process*		
VIT_06s0004g07040	glutaredoxin	−1.08
*Regulation of biological process*		
VIT_00s0684g00020	calmodulin binding IQD20 (IQ-domain 20)	−1.03
*Regulation of molecular function*		
VIT_01s0010g03930	WRKY transcription Factor (VvWRKY03)	−1.39
VIT_10s0003g00870	TCP family transcription factor 4	1.38
VIT_19s0014g03300	NAC domain-containing protein (VvNAC18)	−1.27
VIT_19s0027g00870	NAC domain-containing protein (VvNAC30)	−1.57
VIT_19s0090g00590	MYB domain protein 101	−1.07
*Response to stress*		
VIT_00s0370g00070	epoxide hydrolase	1.27
VIT_01s0150g00450	respiratory burst oxidase protein D (RBOHD)	−1.45
VIT_05s0077g02020	epoxide hydrolase	−1.26
*Secondary metabolic process*		
VIT_00s0724g00010	pinene synthase	−1.01
VIT_06s0004g05670	glutathione S-transferase 25	−1.01
VIT_15s0046g03580	(+)-neomenthol dehydrogenase	1.06
VIT_16s0100g01190	stilbene synthase (VvSTS47)	−1.39
VIT_18s0001g04110	(−)-germacrene D synthase (VvTPS05)	1.10
VIT_18s0001g11430	flavonoid 3-monooxygenase	−1.96
VIT_19s0093g00320	glutathione S-transferase (VvGST1)	−1.65
VIT_19s0135g00180	CYP72A59	−1.10
**1103P vs SO4 at 14 days**		
*Cell wall organization and biogenesis*		
VIT_04s0044g01010	pectinesterase family	1.15
VIT_05s0020g02540	endo-1,3;1,4-beta-D-glucanase precursor	1.06
VIT_09s0002g06380	polygalacturonase GH28	1.12
*Cellular process*		
VIT_15s0048g01680	CYP704G7	−1.35
VIT_15s0048g01690	CYP86A1	−1.28
VIT_18s0001g11490	CYP82C1p	−1.51
VIT_18s0001g11560	CYP82A3	−1.20
*Establishment of localization*		
VIT_01s0011g03020	HAK5 (High affinity K+ transporter 5)	−1.08
VIT_04s0008g04830	ABC Transporter (VvPDR31 - VvABCG61)	−1.49
VIT_04s0023g02530	non-intrinsic ABC protein 12	−1.03
VIT_08s0007g03710	ABC Transporter (VvPDR23 - VvABCG53)	1.10
VIT_09s0002g03550	ABC Transporter (VvPDR3 - VvABCG33)	−1.92
VIT_09s0002g05380	ABC transporter g family pleiotropic drug resistance 12 PDR12	−1.43
VIT_09s0002g05470	ABC transporter g family pleiotropic drug resistance 12 PDR12	−1.57
VIT_09s0018g00900	ABC transporter g family pleiotropic drug resistance 12 PDR12	−1.54
VIT_13s0106g00580	potassium channel AKT1	−1.54
VIT_14s0068g00210	ABC Transporter (VvMDR16 - VvABCB16)	−1.18
VIT_18s0001g10650	ABC transporter g family pleiotropic drug resistance 12 PDR12	−1.22
*Homostatic process*		
VIT_04s0023g02800	glutaredoxin	1.03
*Lipid metabolic process*		
VIT_13s0064g01480	lipoxygenase LOX1	−1.59
VIT_13s0064g01490	lipoxygenase	−1.30
*Regulation of molecular function*		
VIT_01s0127g00640	bHLH	−1.15
VIT_03s0167g00070	putative MADS-box Short Vegetal Phase 5	1.18
*Response to endogenous stimulus*		
VIT_04s0008g06000	VvERF045	1.04
VIT_04s0079g00160	auxin response factor 8	−1.06
*Response to stress*		
VIT_00s0120g00010	peroxisomal biogenesis factor 11 (PEX11C)	−1.09
VIT_00s0370g00060	epoxide hydrolase 2	1.19
VIT_12s0059g02410	peroxidase	1.43
VIT_13s0067g02360	peroxidase, class III	−1.35
*Secondary metabolic process*		
VIT_00s0444g00010	laccase	−1.17
VIT_00s0704g00020	5-alpha-taxadienol-10-beta-hydroxylase	1.04
VIT_00s0731g00010	laccase	−1.18
VIT_00s1212g00020	laccase	−1.33
VIT_03s0063g01590	CYP82C4	1.33
VIT_06s0004g07650	taxadien-5-alpha-ol-O-acetyltransferase	−1.26
VIT_06s0009g03050	flavonoid 3′,5′-hydroxylase	−1.21
VIT_07s0031g01380	ferulate 5-hydroxylase	−1.43
VIT_12s0028g02700	isoflavone methyltransferase	−1.69
VIT_13s0067g00380	pinene synthase	−1.14
VIT_17s0000g05610	isopiperitenol dehydrogenase	1.08
VIT_18s0001g01010	laccase	−1.32
VIT_19s0093g00350	glutathione S-transferase 25	1.09

## Discussion

### 1103P is more tolerant to Mg deficiency than SO4

Physiological and molecular responses to Mg deficiency were widely described in several plant species [11, 42, 43]. The results here presented allowed us to identify the key molecular mechanisms underlying the responses to Mg shortage exhibited by two grapevine genotypes showing a different tolerance to this nutritional disorder. Considering grapevine, no description about the physiological and molecular responses to the shortage of this macronutrient is available, although a different tolerance to Mg deficiency among grapevine rootstocks was reported [38, 44]. Changes in physiological parameters (Figs. 1, 2 and 3) confirmed the appearance of typical symptoms described for other plant species such as chlorosis on older leaves and impairment of carbohydrate partitioning [45, 46]. Interestingly, the Mg-shortage-like changes were more evident for microcuttings of the SO4 (susceptible) rootstock.

The earlier plant response to Mg shortage consists in the impairment of the carbohydrate partitioning between leaves and roots, causing an accumulation of sugars in source leaves and a reduced translocation of them to roots [11]. Our results obtained for SO4 are in line with the increase in leaf glucose and fructose concentrations due to Mg deficiency reported for the first time in bean [13, 14] and later in other species [17, 19, 20, 47–50]. The alteration of carbohydrate partitioning between leaves and roots can cause a decrease in root growth as reported in bean [13, 14]. On the other hand, in other plant species such as sugar beet [19], arabidopsis [20] and rice [17], the Mg deficiency can be responsible for a negative effect on shoot growth as we observed in the susceptible genotypes (Fig. 2b). Verbruggen and Hermans [42] ascribed these contrasting effects on shoots or roots growth to the plant species or to the experimental conditions used for applying Mg shortage (e.g. Mg depletion since germination and/or very young growth stage or plants developed in Mg replete conditions before transfer to Mg deficient solution). Considering these observations, we might hypothesize that our results can be ascribable to the experimental design, in which microcuttings, before the 14-days deficiency period, were first exposed to a diluted NS containing Mg.

The results hereby presented, obtained with microcuttings grown in controlled conditions (i.e. growth chamber, plants in hydroponics), confirmed the difference in the tolerance to Mg deficiency observed in field for these two rootstocks. In both rootstocks, shoot Mg concentration (Fig. 4a) was under the threshold value (about 0.2% of dry weight) recorded for grapevine plants in optimal nutritional conditions and without any deficiency symptoms [51]. However, only the susceptible genotype displayed Mg-shortage responses (Figs. 1, 2 and 3) albeit, under Mg deficiency, shoot and root Mg concentrations were lower in the microcuttings of the tolerant 1103P rootstocks than in the susceptible one (Fig. 4). In grapevine, significant differences in ions bound in the apoplast in a not exchangeable form were reported as influenced by the genotype [52, 53] suggesting the need of an accurate evaluation of Mg distribution in sub-cellular compartments.

### Metabolite profiling suggested a reduced oxidative stress condition in root of 1103P in response to Mg deficiency

It was reported that Mg deficiency could increase the reactive oxygen species (ROS) levels in plant roots [11]. Our metabolite profiling analysis suggests that the tolerant rootstock could better control the oxidative stress maintaining a lower level of ROS in roots through a higher production of antioxidative metabolites, already after 4 days of Mg deficiency. Indeed, roots of 1103P featured a higher concentration of carotenoids, as well as their oxidized forms, and anthocyanins (pelargonidin 3-O-(6-O-malyl)- β-D-glucoside) (Table 2 and Additional file 2: Table S2), which have a potential role in reducing the accumulation of ROS [54, 55]. In addition, Mg deficient 1103P rootstock showed, at both sampling time-points (4 and 14 days), higher levels of the dehydroxyascorbate (Additional file 2: Table S2 and Additional file 3: Table S3), which has antioxidant activity. Consistently, this compound was also reported to increase in *Arabidopsis thaliana* roots after 1 week of Mg deficiency [30]. A stronger capability of 1103P to contrast ROS is also supported by the higher abundance of molybdenum cofactor (Moco) recorded after 4 days (Table 2). Some Moco-dependent enzymes are involved in sulfite (sulfite oxidase and sulfite reductase) [56] and hydrogen peroxide (xanthine dehydrogenase) [57] detoxification. In addition, the lower thiamin and 5′-diphosphate 3′-diphosphate (ppGpp) levels (Table 2) in 1103P further support the hypothesis that root tissues are less prone to oxidative stress. Thiamin levels increases in response to several stresses among which oxidative stress in *Arabidopsis thaliana* [58] and *Zea mays* [59], whilst the plant alarmone seems to play a role in systemic signaling in response to abiotic stimuli [60]. In 1103P, the limited stress-condition of roots is also supported by lower levels of maltose as compared with SO4 (Table 2). Indeed, maltose is involved into the protection of proteins, membranes and photosynthetic electron transport chain; in fact, its levels were shown to increase in *Arabidopsis thaliana* leaf tissues during temperature shock [61]. The response to antioxidative stress in roots caused by Mg shortage appear to be delayed in the susceptible rootstock in comparison to the tolerant one. In fact, other metabolites (3,5-dihydroxyanisole and allylcysteine, Table 2) involved in oxidative stress responses [62, 63] showed higher levels in SO4 than 1103P only after 14 days. Nevertheless, at the same sampling time-point, 1103P produced higher quantity of phosphatidylcholine (Table 2). It was hypothesized that phosphatidylcholine plays a role in root system development of *Arabidopsis thaliana* through phosphatidic acid (PA) signaling [64]. PA is a secondary messenger playing a role in membrane-trafficking, in Ca^2+^ signaling and in the oxidative burst [65].

Other metabolites differentially accumulated in roots under Mg shortage in the two rootstocks are involved into defense responses, cell wall metabolism and gibberellin (GA) metabolism. Regarding the defense responses, we observed a rootstock-specific synthesis of compounds playing a role in these processes. After 4 days, a higher quantity of alkaloids (Table 2) was recorded in 1103P whilst SO4 produced more phytoalexin kauralexin B2 [66], leachianone G (intermediate of sophoraflavanone G synthesis) [67, 68], homomethionine (precursor of glucosinolates) [69] and the allelopathy L-3,4- dihydroxyphenylalanine (L-DOPA) [70]. The analysis of metabolites involved in responses to abiotic and/or biotic stresses, confirmed that the susceptible rootstock (SO4) has a delayed reaction in comparison to the tolerant one (Fig. 6 and Additional file 4: Figure S3). We observed that only at 14 days SO4 accumulated the higher levels of defense compounds (Table 2) such as alkaloids and their precursors, phenolic compounds and their precursors and terpenoids [71–78].

The microcuttings of the tolerant rootstock could respond to the Mg shortage through a positive modulation of monosaccharides (e.g. β-L-arabinopyranose, α-L-arabinofuranose, α-D-xylose, β-D-xylose, Table 2) involved into the synthesis of cell wall xyloglucans and pectins [79]. These results agree with recent reports concerning an increase in arabinose and xylose observed in *Vitis vinifera L. calli* in response to N-, P- and S-deficiency [80]. In undifferentiated and differentiated tissues of grapevine, N and P deficiencies caused a decrease in cellulose that can be compensated, in order to reinforce the cell wall, by an increase in pectin with a lower level of methylesterification and a higher quantity of arabinose-containing polysaccharides, putatively tightly linked to the cell wall [81]. It is thus conceivable that Mg shortage could determine a similar response into cell wall of roots, phenomenon that is more evident in the tolerant genotype. In addition, the higher levels of ferulate measured in Mg deficient 1103P (Table 2) suggested matrix reinforcement through cross-linking caused by esterification with arabinoxylans. This phenomenon was also described for grasses under salt stress [82]. In addition, a decrease of cell wall loosening controlled by gibberellin (GA) in root elongation zone [83] could occur as suggested by a lower level of intermediates (GA12 and methyl-GA9; Table 2) of the biosynthesis of bioactive GA in 1103P [84]. Higher quantity of these intermediates in SO4 roots under Mg shortage can be linked to a significant reduction in S/R ratio (Fig. 2b) in this rootstock.

### Transcriptional analysis reinforces the hypothesis that SO4 has a delayed response to Mg deficiency

The analysis of the differentially expressed transcripts (Additional file 6: Table S6 and Additional file 7: Table S7) in roots under Mg deficiency confirmed the different physiological and metabolic behavior of 1103P and SO4, as mentioned above. Among these responses, the tolerant rootstock exhibited a lower oxidative stress in the first phase of the response to Mg deficiency (e.g. 4 days). This observation is supported by a down-regulation in 1103P roots of a transcript encoding a Respiratory burst oxidase protein D at 4 days (RBOH D). Plant RBOHs are involved into the production reactive oxygen intermediates (ROI) [85]. In addition, after 14 days we observed in 1103P roots the down-regulation of the peroxisomal biogenesis factor 11 transcript (PEX11C; Table 4). It was reported that OsPEX11 play a role in response to salt stress through the modulation of cation transporters and increasing the activity of antioxidant enzymes [86]. Nevertheless, we did not observe a clear genotype-specific transcriptional behavior of genes encoding enzymes (e.g. epoxide hydrolase, EH; GRX; GST; lipoxygenase, LOX; peroxidase, POX; Table 4 and Fig. 8) involved into the responses to oxidative stress at 4 and 14 days of Mg deficiency [87–90]. On the other hand, we observed a lower level of phenolics in 1103P root tissues under Mg shortage in particular at 14 days (Table 2). Phenolic compounds are involved in the non-enzymatic antioxidative responses in plant tissues [91]. Transcriptional data are in line with this assumption, showing a main down-regulation of transcripts encoding for enzymes involved into phenolic biosynthesis such as flavonoid 3-monooxygenase (F3H), isoflavone methyltransferase (IMT), ferulate 5-hydroxylase (F5H), flavonoid 3′,5′-hydroxylase (F3’5’H), laccase and stilbene synthase (STS) and transcripts encoding cytochrome P450 proteins (CYP) (Table 4, Additional file 8: Figure S8). Considering terpene biosynthesis, our transcriptional analysis (Table 4) suggested that 1103P, compared to SO4, selectively modulated transcripts involved into the biosynthesis of some monoterpenes, sesquiterpene and diterpene in response to Mg shortage. A lower level of monoterpene was measured in 1103P roots, in particular at 14 days (Table 2). At the same time, a strong repression of transcripts encoding ATP-binding cassette (ABC) transporters was measured (Table 4). ABC transporters could play a role into the export of secondary metabolites such as terpenoids, alkaloids, and phenolics to the cell surface and/or in the accumulation into the vacuole of their non-toxic precursors [92]. This transcriptional behavior can explain the lower synthesis of these classes of compounds in 1103P at 14 days (Table 2).

Considering the cell wall metabolism, transcripts encoding cellulose synthase (CEL), endo-1,3;1,4-beta-D-glucanase (GH), pectinesterese (PE) and polygalacturonase (PG) were up-regulated in 1103P as compared with SO4 mainly at 4 days (Table 4). These results partially fit with the previously hypothesized changes in cell wall structure consisting in a decrease of cellulose content and its stiffness through an increase in pectin with a lower level of methylesterification and a higher quantity of arabinose-containing polysaccharides putatively tightly linked to the cell wall as described in response to mineral deficiency in grapevine [81]. Although the N and P deficiencies caused in grapevine a decrease in cellulose, it was reported an up-regulation of transcripts for CELs [81]. This lack of correlation could also occur in the response to Mg shortage of 1103P, exhibiting higher levels of expression of CELs and GH genes involved in cellulose biosynthesis (Table 4). The up-regulation of PE transcripts was in line with the hypothesized increase in methylesterification of pectin [81].

Considering cellular responses after the perception of low Mg, it was reported an increase of ROS and cytosolic calcium (Ca^2+^) concentration during the elongation of root hairs in response to Mg shortage in *Arabidopsis* plants [31, 93]. The down-regulation of a transcript encoding a Calmodulin binding IQD20 protein (Table 4) in 1103P in comparison with SO4 at 4 days further supports the hypothesis that the tolerant genotype can counteract more efficiently the oxidative stress caused by Mg deficiency by limiting ROS development and modifying the root apparatus. In fact, IQD proteins could play a role in auxin and Ca^2+^ signaling in order to regulate plant growth and development [94]. In addition, the repression of an auxin response factor (ARF; Table 4) was in line with this hypothesis since it was proved that the expression of IQD genes is regulated by auxin though ARFs [94].

The processes linked to Mg uptake and redistribution could be a crucial point for the different capability of plants to cope with Mg shortage. The transporters MRS2/MGT have been often related to Mg uptake and, in particular, some members (i.e. AtMGT6) of the family have been suggested to be involved in the acquisition of the nutrient in shortage condition [95]. However, in Mg deficiency we did not observe a differential expression of MRS2/MGT transcripts between the two rootstocks. Nevertheless, a protein belonging to this transporter family (Additional file 7: Table S7, *VIT_07s0031g02820*) was shown to be up-regulated in 1103P plants grown in the presence of Mg for 14 days. This grapevine transcript encodes a protein showing the highest homology with the AtMRS2–10 (AtMGT1; AT1G80900.1, 40% identity) that is described as a high affinity Mg^2+^ transporter localized on plasma membrane and expressed in particular in root hair and in elongation zone [95, 96]. However, as recently highlighted [96], the Mg transport mediated by this protein is induced by Al toxicity but not by Mg deficiency. Indeed, other transport mechanisms could be involved in the modulation of Mg uptake in response to Mg deficiency [96]; it was in fact reported that the high-affinity K^+^ transporter OsHKT2;4 can accomplish a Mg^2+^ and Ca^2+^ transport that is impaired by K^+^ oversupply [97]. Interestingly, we observed the up-regulation of a HKT2 transcript in roots of 1103P as compared to SO4 under 4-day Mg deficiency (Table 4) and, at 14 days, the down-regulation of a transcript encoding a different putative HKT (Table 4), which shows the highest amino acid homology with AtHKT5 (AT4G13420.1, 60%). The AtAKT1 channel, AtHAK5 and AtKUP7 transporters are considered the major Arabidopsis components for K^+^ uptake at the root-soil interface [98]. In addition, a transcript putatively involved in the intracellular K^+^ homeostasis (e.g. Cation/H^+^ exchanger) [99] and a transcript encoding for a K^+^ channel (AKT1) mediating the low-affinity transport in plant roots [100] were repressed in 1103P at 4 and 14 days of treatment, respectively (Table 4). Overall, these results suggest that 1103P could positively modulate the component putatively involved in Mg uptake in 1103P (HKT2, *VIT_11s0103g00010*) with a concomitant down-regulation of the genes putatively responsible for K^+^ uptake and homeostasis.

Our results underlined a differential expression between the two rootstocks under Mg shortage of transcripts encoding several transcription factors that could be involved in the regulation of the different response to Mg deficiency. At both times, transcripts encoding transcription factors were mainly down-regulated in 1103P as compared with SO4 (Additional file 6: Table S6 and Additional file 7: Table S7). In particular, at 4 days we observed a repression of transcripts encoding a MYB protein showing amino acid homology to AtMYB101 (81% identity) among the *Arabidopsis thaliana* MYBs. *AtMYB101* together with *AtMYB33* and *AtMYB65* was reported to be negatively regulated by *miR159* that is described as a post transcriptional negative regulator of primary root growth in *A. thaliana* [101]. This transcriptional behavior suggests a different response of root apparatus to Mg deficiency between 1103P and SO4. A similar transcriptional behavior was recorded for one and two transcripts encoding a WRKY protein and two NAC proteins respectively (Table 4). The plant transcription factors WRKYs regulates in plants the responses to several biotic and abiotic stresses, among which nutritional stress as tolerance to low phosphate (Pi) condition [102, 103]. The grapevine WRKY transcript encodes a protein showing the highest homology among the *A. thaliana* a WRKYs with AtWRKY75 (84% amino acid identity), a negative regulator of root growth, whose expression was induced under phosphate (Pi) starvation [104]. Concerning NAC proteins, it was reported their involvement in responses to several abiotic stress [105]. Only a TEOSINTE BRANCHED 1, CYCLOIDEA, PCF1 (TCP) transcripts was more expressed at 4 days of deficiency in 1103P as compared to SO4 (Table 4). Members of TCP family are involved into control of plants growth in response to environmental signals such as red to far-red light ratio, high light stress, salt stress or abundance of nutrients (e.g. nitrate) [106]. After 14 days of deficiency other genes encoding transcription factors could play a role in the differential regulation of the late responses to this stress condition between the two grapevine roots. We observed in 1103P a minor expression level of a basic/helix-loop-helix (bHLH) whilst an opposite transcriptional profile was recorded for transcripts encoding an ethylene transcription factor (*VvERF45*) and a MADS-box protein (Table 4).

## Conclusion

Our results allowed obtaining for the first time a picture of the different molecular responses exhibited by two contrasting genotypes with respect to the tolerance towards Mg shortage. In particular, data here presented clearly indicate that the ability of 1103P to tolerate Mg deficiency mainly relies on the early control of ROS level, thus reducing the oxidative stress in roots. In comparison with SO4, the molecular events playing a role in these responses are in fact faster both in terms of metabolites and transcripts involved. Although the data concerning the capability of acquiring cation nutrients more efficiently by the tolerant rootstock are somehow difficult to be interpreted, it seems most likely that the tolerance mechanism might rely on a tight control of cations ratios and homeostasis. Further research is indeed necessary to understand if the responses here highlighted could be generalized also to other plant species.

## Methods

### Plant material and sampling

Two-week-old grapevine in vitro propagated microcuttings (provided by Vitroplant s.r.l.) of SO4 and 1103P rootstocks were hydroponically grown into 2-L plastic pots for two weeks using a dilute nutrient solution (NS) containing: 200 μM Ca(NO_3_)_2_, 50 μM MgSO_4_, 70 μM K_2_SO_4_, 10 μM KCl, 10 μM KH_2_PO_4_, 0.1 μM H_3_BO_3_, 0.05 μM MnSO_4_, 0.02 μM CuSO_4_, 0.05 μM ZnSO_4_, 0.001 μM (NH_4_)_6_Mo_7_O_24_ and 10 μM Fe-EDTA. The pH of the solution was adjusted to 6.0 ± 0.1 with KOH. The NS was changed every week and the microcuttings were grown in a growth chamber (Photosynthetically Active Radiation, PAR, 200 μmol m^− 2^ s^− 1^, 16/8 h day/night regime, temperature 24 °C). After two weeks, for the Mg starvation experiment a NS with the following composition was set-up: 1000 μM Ca(NO_3_)_2_, 350 μM K_2_SO_4_, 50 μM KCl, 50 μM KH_2_PO_4_, 0.5 μM H_3_BO_3_, 0.25 μM MnSO_4_, 0.1 μM CuSO_4_, 0.25 μM ZnSO_4_, 0.005 μM (NH_4_)_6_Mo_7_O_24_ and 50 μM Fe-EDTA. For both rootstocks, half of the microcuttings were grown in this new NS containing 250 μM MgSO_4_ (+Mg) and the other half in a Mg-free NS in which MgSO_4_ was replaced with an equivalent mole of CaSO_4_ (−Mg). Three independent experiments (biological replicates) were performed growing 24 microcuttings (6 for each pot) for each treatment (SO4 + Mg, SO4 -Mg, 1103P + Mg and 1103P -Mg). For each biological replicate, root tissues from six plants were collected and pooled for each sample (SO4 + Mg, SO4 -Mg, 1103P + Mg and 1103P -Mg) after 4 and 14 days of growth. Samples were ground with liquid nitrogen and used for microarray analysis and Real time RT-PCR analysis, GC-MS analysis, UPLC-MS analysis.

Shoot to root (S/R) ratio and SPAD index were determined at the end (14 days) of each independent experiment using four plants (technical replicates). S/R was calculated measuring the fresh root and shoot weights of each plant. For the same plant a mean SPAD index was calculated using the SPAD values of all the leaves. For each leaf the SPAD value was expressed as the mean of five measurements performed using a SPAD-502 Plus Chlorophyll meter® (Konica Minolta). For each of the three independent experiments (biological replicates, *n* = 3) the mean value of S/R and SPAD index was calculated using the data its technical replicates. Data were expressed as mean ± SEM (*n* = 3, biological replicates).

### Soluble sugars quantification

Shoot total soluble sugars were quantified using the Sucrose/D-Fructose/D-Glucose Assay kit (Megazyme, USA) according to the manufacturer’s protocol using leaf tissues of three plants (technical replicates) for each independent experiment (biological replicates) sampled at the end of the growth (after 14 days). For each of the three independent experiments (biological replicates, n = 3) the mean value was calculated using the data of their technical replicates. Data were expressed as mean ± SEM (n = 3, biological replicates).

### Mg quantification

Magnesium concentrations were determined through ICP-MS analysis using three plants (technical replicates) for each independent experiment (biological replicates) sampled at the end of the growth (after 14 days). Roots and shoots were separated, washed three times in deionized water and two time with ultrapure water (milliQ, 18.2 MΩ cm) and dried in oven at 60 °C for 72 h. Samples (5 mg) were weighted and digested in a TFM microsampling insert using 250 μl of 69% ultrapure HNO_3_. Three inserts were put into 100-ml oven vessel containing 10 ml of water (milliQ, 18.2 MΩ cm) and 1 ml of 30% H_2_O_2_. In addition, 5 mg of the following reference material were digested: NIST 1515 (apple leaves). Digestion reaction was carried out using a microwave oven (Milestone StartD® microwave). A 20-min ramping period was used to reach a digestion temperature of 180 °C, which thereupon was maintained for 20 min. Samples were then diluted with water (milliQ, 18.2 MΩ cm) to a final concentration of 2% HNO_3_. Sample Mg concentrations were determined by using the Agilent 7500cx ICP-MS (Agilent). The instrument was tuned using tuning solution (Agilent tuning solution 1 ppb) in a standard mode checking the sensitivity of masses ^7^Li, ^89^Y, and ^205^Tl and the oxide and double charged ion levels (< 2%). Mg was quantified using a Mg standard solution (Romil PrimAg-plus). For each of the three independent experiments (biological replicates, n = 3) the mean value was calculated using the data of their technical replicates. Data were expressed as mean ± SEM (n = 3, biological replicates).

### GC/MS metabolomic analysis

An amount of 150 mg of grinded root tissue was weighted in 2 mL tube and used for extraction in 80% methanol. After vortexing, the samples were sonicated for 5 min and centrifuged at 11000 g for 10 min at 4 °C. The following GC/MS metabolomic analysis was carried out according to Lisec et al. [107]. A volume of 200 μL of supernatant of each sample was added with 5 μL of internal standard (norvaline 2 mM) and then dried in a SpeedVac™ (Thermo Scientific, Waltham, MA, USA). For derivatization, each sample was added with 3 μL of a second internal standard (myristic acid 10 mM), then with 60 μL of methoxyamine in pyridine (20 mg/ml), incubated at 37 °C with shaking for 30 min and then without shaking for 3 h, in order to protect carbonyl groups. Thereafter, the samples were transferred into vials and added with 90 μL of *N*-methyl-*N*-(trimethylsilyl) trifluoroacetamide (MSTFA) with 1% trimethylchlorosilane, as derivatization agent.

The tubes were incubated in ThermoMixer at 400 rpm, for one hour at 55 °C and then transferred into vials to be analyzed.

An Agilent 6890 gas chromatograph equipped with a 30 m DB-5MS capillary column and coupled to an Agilent 5977 quadrupole mass spectrometer was used. Derivatized extracts (1 μl) were injected in splitless mode (250 °C), using helium as carrier gas (1 ml/min). A GC oven program, starting at 100 °C (hold for 2 min) up to 325 °C at 10 °C/min, was adopted. A fatty acids methyl ester mixture was used for retention time locking purposes (FAME mix, Agilent Technologies). Features were deconvoluted using the software “Unknown Analysis” (Agilent Technologies) and identification was based on spectral comparison against the commercially available database Fiehn Library (Agilent Technologies).

### UHPLC-ESI/QTOF-MS metabolomic analysis

An aliquot of the extract produced for GC/MS was filtered through a 0.22 μm cellulose membrane and transferred to an amber vial for a complementary untargeted metabolomic analysis. Plant metabolites were screened using UHPLC chromatography coupled to a quadrupole-time-of-flight mass spectrometer (UHPLC/ QTOF-MS) through a JetStream Electrospray ionization system. In detail, a 1290 series liquid chromatograph, equipped with a binary pump, was interfaced to a G6550 iFunnel mass spectrometer (all from Agilent Technologies).

Instrumental conditions were optimized for plant metabolomics in previous experiments [108]. Briefly, chromatographic separation was achieved using a mixture of methanol and water as mobile phase and an Agilent Zorbax Eclipse-plus column (75 × 2.1 mm i.d., 1.8 μm). The gradient started from 5 to 90% methanol within 35 min, with a flow of 220 μL min^− 1^. Injection volume was 3.5 μL and the QTOF was run in positive polarity and SCAN mode (100–1200 m/z^+^ range, 0.8 spectra s^− 1^). Compounds deconvolution, mass and retention time alignment, as well as filtering (mass accuracy < 5 ppm, filter-by-frequerncy with 100% of detection within at least one condition) were carried out in Profinder B.06 (Agilent Technologies). Compounds annotation was based on accurate mass, isotope spacing and isotope ratio, versus the database exported from PlantCyc 9.5 [109]. Molecular features were subjected to a recursive analysis workflow using Mass Profiler Professional B12.06 (from Agilent Technologies) after the initial deconvolution. The peak area of each compound identified after recursive analysis with an identification score above 85/100 was extracted from the total ion current and exported for statistics and data interpretation.

Therefore, compounds identification was carried out as Level 2 (putatively annotated compounds), according to COSMOS Metabolomics Standards Initiative (http://cosmos-fp7.eu/).

Interpretation of either GC/MS and UHPLC-ESI/QTOF-MS metabolomics was carried out in Mass Profiler Professional B.12.06 (Agilent Technologies). Compounds’ abundance was normalized at the 75th percentile and baselined to the median. Thereafter, multivariate ANOVA (*P* < 0.05, Benjamini–Hochberg multiple testing correction) and fold-change analysis (cut-off = 2) were combined into volcano plots.

### Microarray analysis

The total RNA was extracted from 100 mg of root tissues using the Spectrum Plant Total RNA kit (Sigma-Aldrich). RNA quality and quantity were verified using a Nanodrop 2000 instrument (Termo Scientific) and a Bioanalyzer Chip RNA 6000 series II (Agilent Technologies). The cDNA synthesis, labelling, hybridization and washing procedures were carried out according to the NimbleGen Arrays User’s Guide (version 3.2). Each sample was hybridized to one sub-array of a NimbleGen microarray 090818 Vitis exp. HX12 (Roche, NimbleGen; GPL13936) containing probes targeted to 29,549 predicted grapevine transcripts. The microarrays were scanned using an Axon GenePix 4400A (Molecular Devices) at 532 nm (Cy3 absorption peak) and GenePix Pro7 software (Molecular Devices) following the manufacturer’s instructions. A NimbleScan v2.5 software (Roche) was used for images analyses. This software produces Pair Files containing the raw signal intensity data for each probe and Calls Files with normalized expression data (quantile normalization) [110] derived from the probe summarization, performed through RMA analysis [111]. Analysis of normalized data (Calls Files) was performed using the open source software of the Bioconductor project [112] (http://www.bioconductor.org) with the statistical R programming language [113] (http://www.r-project.org). Differentially expressed probes were identified by Linear Models for MicroArrays [40] using LIMMA package and applying Bayesian correction, adjusted *P*-value of 0.05 and a |Log_2_(T/C)| ≥ 1. All microarray expression data are available at the GEO (http://www.ncbi.nlm.nih.gov/geo) under the series entry (GSE117849). Transcript annotation of the V1 version is reported by Fasoli et al. [114].

### Real time RT-PCR analysis

Microarray data were validated by quantitative RT-PCR experiments analyzing the expression profile of 8 transcripts (Additional file 9: Table S8). Primer sequences were reported in Additional file 9: Table S9. Transcripts encoding tubulin beta chain (*VIT_08s0040g00980*) and elongation factor 1-alpha (*VIT_06s0004g03220)* were used to normalize data. DNA traces were removed with DNase I treatment (RQ1 RNase-Free DNase, Promega) according to the manufacturer’s procedure from total RNA samples used in microarray experiments. Reverse transcription reactions were performed using 1 μg of total RNA and the ImProm-II Reverse Transcriptase (Promega) and Real-time RT-PCR reactions were performed using FastSYBR® Green Master Mix (Applied Biosystems) according to the manufacturer’s protocols. The reactions were performed using a StepOnePlus™ (Applied Biosystems) with a final volume of 10 μL, the primer concentration of 350 nm and 1 μL of a 1:4 solution of cDNA. The thermal profile was 95 °C for 20s, 40 cycle of 95 °C for 3 s and 60 °C for 30s. PCR reaction efficiencies were calculated with the LinRegPCR program [115]. For each transcript, two mean normalized expression values (MNE) [116] were calculated using separately the two housekeeping transcripts for each sample (SO4 + Mg, SO4 -Mg, 1103P + Mg and 1103P -Mg). A final mean normalized expression value was calculated using a geometric mean of the two normalized expression values obtained for each transcript [117] and each sample. These normalized expression values were than used to calculated fold change (FC) values between samples (Additional file 7: Table S7).

### MapMan analysis

Differentially abundant metabolites and differentially expressed transcripts were mapped on biotic stress overview and secondary metabolism overview using MapMan tool [118] (https://mapman.gabipd.org/). Concerning metabolites, a mapping file (Additional file 10: Table S10) was built assigning a bin code at each metabolite using information retrieved from KEGG (KEGG compound) (https://www.genome.jp/kegg/) and PlantCyc [109] databases. In the case of transcripts, the analysis was carried out using the mapping file for the grapevine Nimblegen chip (vvi_Nimblegen_probe_2018-05-25_mapping.txt) (http://protein.gomapman.org/).

## Additional files

Additional file 1: **Table S1.** Metabolites differentially abundant in roots between 1103P and SO4 at 4 and 14 days of growth in presence (+Mg) and absence (-Mg) of Mg identified by the comparison of profile obtained by QTOF and GC-MS analyses. Description, p(Corr) and fold change (FC) were reported. (XLSX 32 kb)

Additional file 2: **Table S2.** List of metabolites differentially abundant in roots at 4 days between 1103P and SO4 depending on the growth condition. Metabolites specifically modulated at 4 days in presence (+Mg) and absence (-Mg) of Mg and modulated between the two genotypes in both nutritional conditions. Identity of compound, p(Corr) and FC were reported. (XLSX 16 kb)

Additional file 3: **Table S3.** List of metabolites differentially abundant in roots at 14 days between 1103P and SO4 depending on the growth condition. Metabolites specifically modulated at 14 days in presence (+Mg) and absence (-Mg) of Mg and modulated between the two genotypes in both nutritional conditions. Identity of compound, p(Corr) and FC were reported. (XLSX 22 kb)

Additional file 4: **Table S4.** More and less abundant metabolites in 1103P relative to SO4 at 4 and 14 days; metabolites were grouped in main chemical classes. **Figures S1-S3**. (PDF 801 kb)

Additional file 5: **Table S5.** Differentially expressed transcripts identified by each comparison of transcriptional profiles. Probe ID, adjusted *p*-value, Log_2_(ratio) and Functional annotation were reported for each transcript. (XLSX 163 kb)

Additional file 6: **Table S6.** List of transcripts differentially expressed in roots at 4 days between 1103P and SO4 depending on the growth condition. Transcripts specifically modulated at 4 days in presence (+Mg) and absence (-Mg) of Mg and modulated between the two genotypes in both nutritional conditions. Probe ID, adjusted *p*-value, Log_2_(ratio), Functional annotation and Gene Ontology term were reported for each transcript. (XLSX 87 kb)

Additional file 7: **Table S7.** List of transcripts differentially expressed in roots at 14 days between 1103P and SO4 depending on the growth condition. Transcripts specifically modulated at 14 days in presence (+Mg) and absence (-Mg) of Mg and modulated between the two genotypes in both nutritional conditions. Probe ID, adjusted *p*-value, Log_2_(ratio), Functional annotation and Gene Ontology term were reported for each transcript. (XLSX 85 kb)

Additional file 8: **Figures S4-S8**. (PDF 958 kb)

Additional file 9: **Table S8.** Real-time RT-PCR of a set of transcripts differentially expressed between roots of 1103P and SO4 at 4 and 14 days of growth in presence (+Mg) and absence (-Mg) resulted differentially expressed by microarray analysis. Probe ID, V1 transcript ID, description, microarray fold change (FC) value and RT-PCR data (means ± SE of three biological replicates) were reported. **Table S9.** Sequence of forward and reverse primers used in Real-time RT-PCR experiments. Probe ID, V1 transcript ID, description, 5’-3’ nucleotide sequence of forward and reverse primers were reported. (PDF 1310 kb)

Additional file 10: **Table S10.** Mapping file of the differentially abundant metabolites identified at 4 and 14 days used to carry out the MapMan analyses. Bin code, name, identifier and type were reported for each metabolite. (XLSX 11 kb)
