# Supplementary material for: The different tolerance to magnesium deficiency of two grapevine rootstocks relies on the ability to cope with oxidative stress
Source: BMC Plant Biol. 2019 Apr 16;19:148. doi: 10.1186/s12870-019-1726-x (PMC6469136; doi:10.1186/s12870-019-1726-x)
Supplement: Supplementary file 4 — Table S4. More and less abundant metabolites in 1103P relative to SO4 at 4 and 14 days; metabolites were grouped in main chemical classes. Figures S1-S3. (PDF 801 kb) [file 12870_2019_1726_MOESM4_ESM.pdf]

Table S4

Metabolites modulated only in the comparison 1103P -Mg *vs* SO4 -Mg; 4 days		
Compound	p (Corr)	FC
**alkaloids**		
more abundant in 1103P *vs* SO4		
(S)-corytuberine	0.035	10080.12
1,2-dehydroreticulinium	0.035	10080.12
norephedrine	0.036	5055.84
**amino acids or their derivatives**		
more abundant in 1103P *vs* SO4		
3-O-methyldopa	0.000	6188.33
N,N-dihydroxy-L-tyrosine	0.036	1877.57
less abundant in 1103P *vs* SO4		
aspartic acid 2	0.000	-16.00
ethionine	0.016	-7.34
homomethionine	0.016	-7.34
L-dopa	0.000	-32643.51
**aromatic compounds**		
less abundant in 1103P *vs* SO4		
biphenyl-2, 3-diol	0.044	-4.32
**carbohydrates**		
more abundant in 1103P *vs* SO4		
aldehydo-D-arabinose	0.000	23691.90
aldehydo-D-ribose	0.000	23691.90
aldehydo-D-xylose	0.000	23691.90
aldehydo-L-arabinose	0.000	23691.90
alpha;-D-ribofuranose	0.000	23691.90
alpha;-D-xylopyranose	0.000	23691.90
alpha;-L-arabinofuranose	0.000	23691.90
beta;-D-ribofuranose	0.000	23691.90
beta;-D-xylopyranose	0.000	23691.90
beta;-L-arabinopyranose	0.000	23691.90
D-ribulose	0.000	23691.90
D-xylulose	0.000	23691.90
L-ribulose	0.000	23691.90
pentose-ring	0.000	23691.93
less abundant in 1103P *vs* SO4		
maltohexaose	0.000	-287939.56

N-acetyl-beta;-glucosaminylamine	0.000	-2.84
**cofactors**		
more abundant in 1103P *vs* SO4		
molybdenum cofactor	0.044	7.38
less abundant in 1103P *vs* SO4		
7,8-didemethyl-8-hydroxy-5-deazariboflavin	0.038	-7.48
**enzyme inhibitors**		
more abundant in 1103P *vs* SO4		
2,4-diamino-6-methyl-5,3'-(3-nitrophenoxy)prop-1'-yloxypyrimidine	0.037	5.76
disulfiram	0.000	40.87
isoleucine tetrazole	0.000	16.00
**gibberellins**		
less abundant in 1103P *vs* SO4		
gibberellin A12	0.000	-22.72
methyl gibberellin A9	0.000	-22.72
**lipids**		
more abundant in 1103P *vs* SO4		
1-18:3-2-16:0-digalactosyldiacylglycerol	0.037	5835.99
dipalmitoyl phosphatidate	0.036	3.38
**nucleic acid components**		
more abundant in 1103P *vs* SO4		
2'-deoxyguanosine	0.002	8.06
adenosine	0.002	8.06
less abundant in 1103P *vs* SO4		
pppGpp	0.000	-131.55
**phenylpropanoids**		
more abundant in 1103P *vs* SO4		
ferulate	0.000	18227.24
pelargonidin 3-O-(6-O-malyl-beta;D-glucoside)	0.000	16.00
less abundant in 1103P *vs* SO4		
leachianone G	0.037	-7.82
p-coumaroyltyramine	0.002	-7.61
**terpenoids**		
more abundant in 1103P *vs* SO4		
1'-hydroxy-gamma;-carotene	0.035	41946.68
beta;-carotene 15,15' epoxide	0.035	41946.68
beta;-cryptoxanthin	0.035	41946.68
epsilon;,epsilon;-carotene-3-diol	0.035	41946.68
less abundant in 1103P *vs* SO4		
kauralexin B2	0.000	-22.72
**vitamins**		

less abundant in 1103P *vs* SO4		
thiamin	0.035	-8.64
**Metabolites modulated only in the comparison 1103P -Mg *vs* SO4 -Mg; 14 days**		
Compound	p (Corr)	FC
**alkaloids**		
less abundant in 1103P *vs* SO4		
(R)-N-methylcoclaurine	0.041	-28523.96
(S)-N-methylcoclaurine	0.041	-28523.96
S-cheilanthifoline	0.000	-16.00
senecionine	0.000	-16.00
**amino acids or their derivatives**		
more abundant in 1103P *vs* SO4		
S-carbamylcysteine	0.000	1424.28
less abundant in 1103P *vs* SO4		
4-guanidinobutyraldehyde	0.034	-46.18
allylcysteine	0.044	-4892.33
**aromatic compounds**		
more abundant in 1103P *vs* SO4		
4-nitrophenol	0.000	16.00
less abundant in 1103P *vs* SO4		
L-arogenate	0.000	-2.51
puromycin	0.036	-2.60
**carboxy acids**		
more abundant in 1103P *vs* SO4		
glyoxylic acid	0.000	16.00
**coenzyme A-activated compounds**		
more abundant in 1103P *vs* SO4		
3-hydroxyisovaleryl-CoA	0.000	16.00
propanoyl-CoA	0.000	2.03
**glucosinolates**		
more abundant in 1103P *vs* SO4		
3-sinapoyloxypropylglucosinolate	0.000	16.00
S-methyl-5-thio-D-ribose	0.041	3207.84
**lipids**		
more abundant in 1103P *vs* SO4		
1-18:1-2-18:3-phosphatidylcholine	0.000	46713708.00
1-18:2-2-18:2-sn-glycerol-3-phosphocholine	0.000	46713708.00
**organosulfur compounds**		
less abundant in 1103P *vs* SO4		
4-methyl-5-(beta;-hydroxyethyl)thiazole	0.044	-4892.33
**organometallic compounds**		
more abundant in 1103P *vs* SO4		
chlorophyllide a	0.000	2.94

**phenylpropanoids**		
more abundant in 1103P *vs* SO4		
feruloyl-CoA	0.000	16.00
less abundant in 1103P *vs* SO4		
3,5-dihydroxyanisole	0.011	-4.45
4-hydroxycinnamic acid	0.000	-16.00
isoliquiritigenin 4'-glucoside	0.008	-3.84
p-coumaroyltyramine	0.003	-11.51
**terpenoids**		
more abundant in 1103P *vs* SO4		
4,9,13-trimethyltetradeca-2,4,6,8,10,12-hexaene-1,14-dial	0.017	8299.46
less abundant in 1103P *vs* SO4		
(-)-menthol	0.000	-39919.66
(+)-isomenthol	0.000	-39919.66
(+)-neoisomenthol	0.000	-39919.66
(+)-neomenthol	0.000	-39919.66
(S)-(-)-citronellol	0.000	-39919.64
loganin	0.004	-2.49
**vitamin**		
less abundant in 1103P *vs* SO4		
9-mercaptodethiobiotin	0.000	-4.68

Figure S1

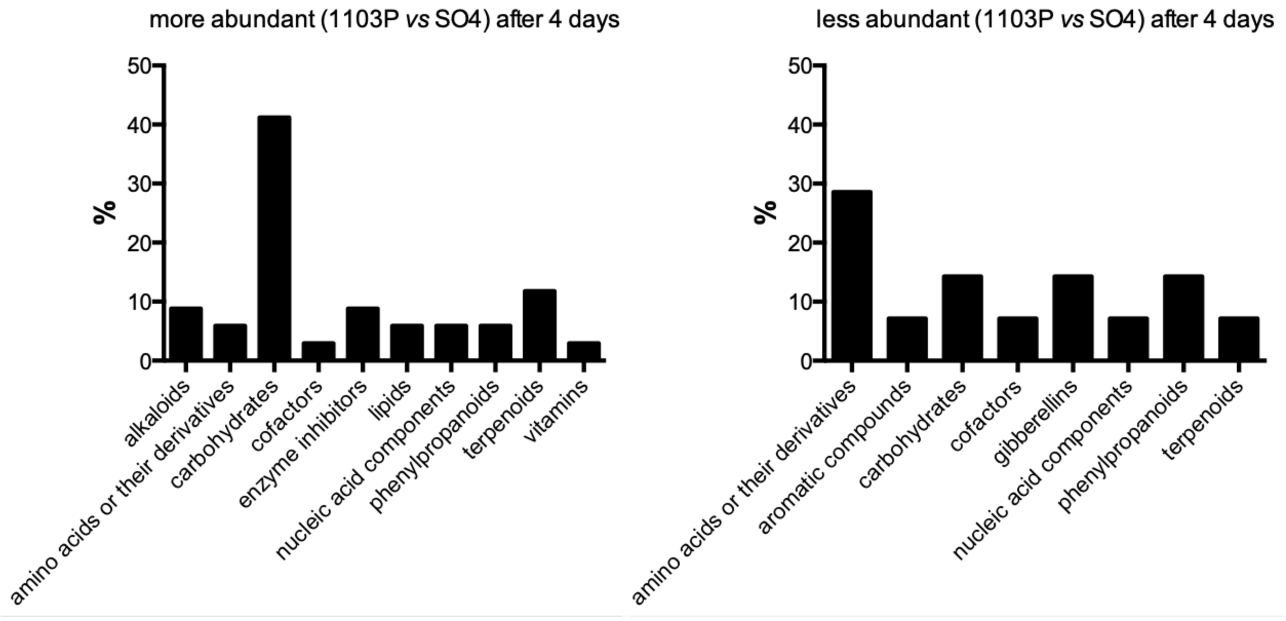

Figure S1. Distribution in main chemical classes of metabolites more abundant (34) and less abundant (14) in 1103P relative to SO4 after 4 days of growth without Mg (-Mg). Metabolites were classified using information retrieved from the PlantCyc database [109].

Figure S2

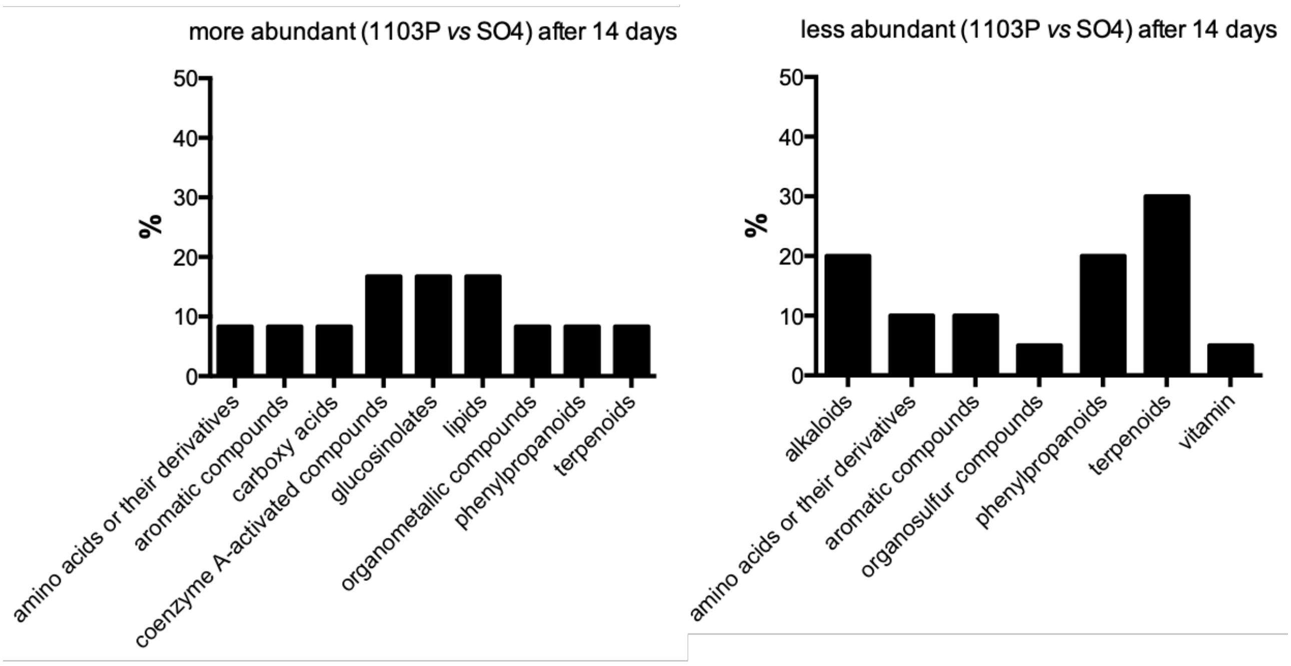

Figure S2. Distribution in main chemical classes of metabolites more abundant (12) and less abundant (20) in 1103P relative to SO4 after 14 days of growth without Mg (-Mg). Metabolites were classified using information retrieved from the PlantCyc database [109].

Figure S3

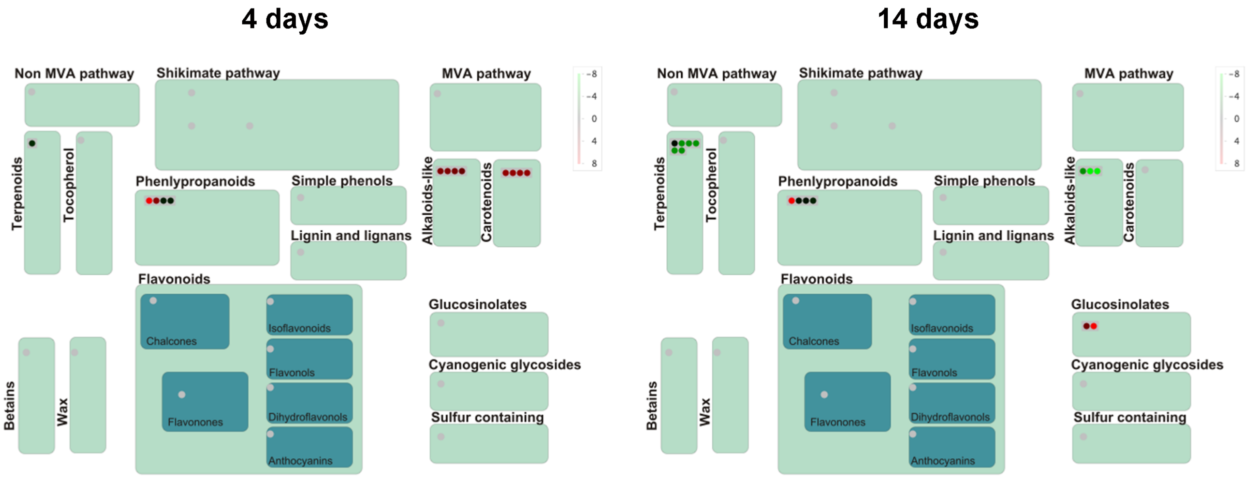

Figure S3. MapMan overview display of differentially abundant metabolites 1103P and SO4 concerning the secondary metabolism. The Log_{10}(ratio) is shown by the color scale (green indicates a decrease and red an increase in metabolite abundance between 1103P and SO4). The analysis was carried out using MapMan software [123].
