# Supplementary material for: The different tolerance to magnesium deficiency of two grapevine rootstocks relies on the ability to cope with oxidative stress
Source: BMC Plant Biol. 2019 Apr 16;19:148. doi: 10.1186/s12870-019-1726-x (PMC6469136; doi:10.1186/s12870-019-1726-x)
Supplement: Supplementary file 8 — Figures S4-S8. (PDF 958 kb) [file 12870_2019_1726_MOESM8_ESM.pdf]

Figure S4

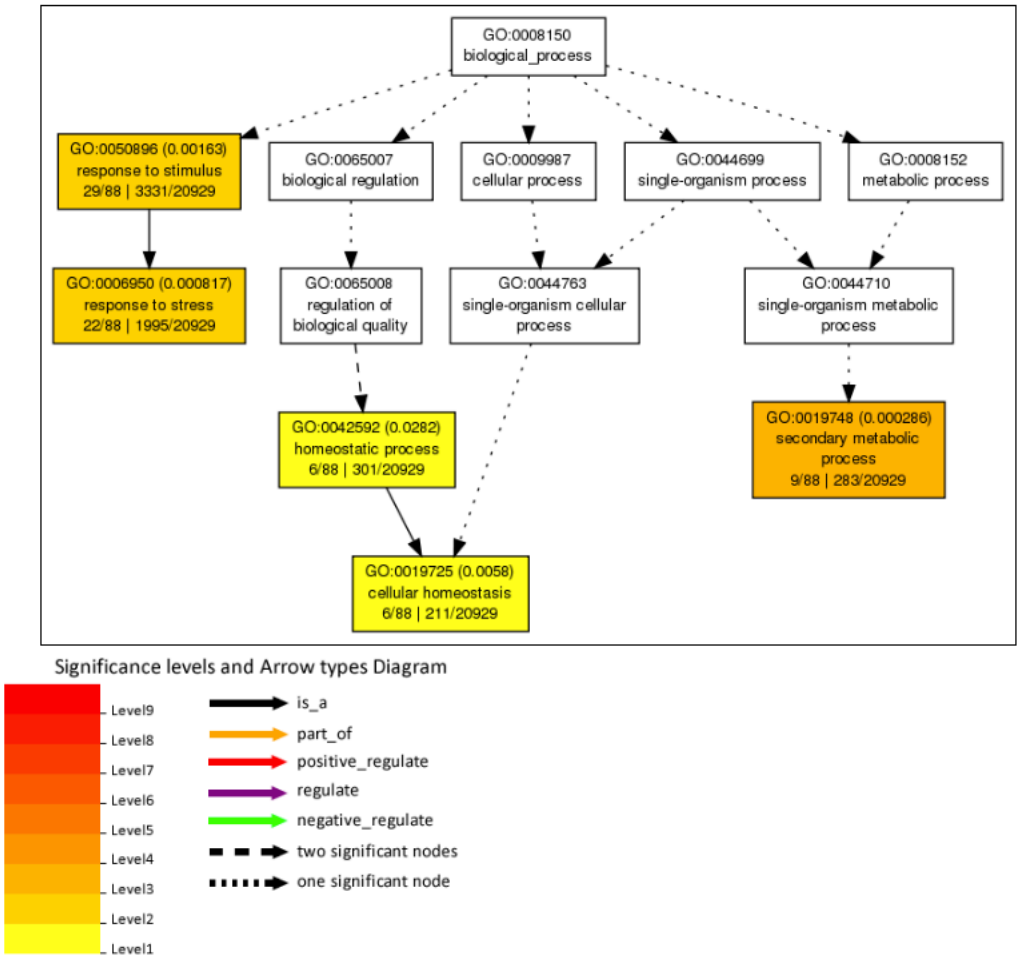

Figure S4. Results of Singular Enrichment Analysis (SEA) for the 88 out of 115 up-regulated transcripts between 1103P and SO4 grown in absence of Mg (-Mg) for 4 days with GO terms; the analysis was performed using agriGO v2.0 [126] using the locus ID (Gramene Release 5.0) as reference, Fisher as statistical test method, Yekutieli (FDR under dependency) as Multi-test adjustment method, 0.05 as significant level, 5 as minimum number of mapping entries and Plant GO slim as Gene Ontology type.

Figure S5

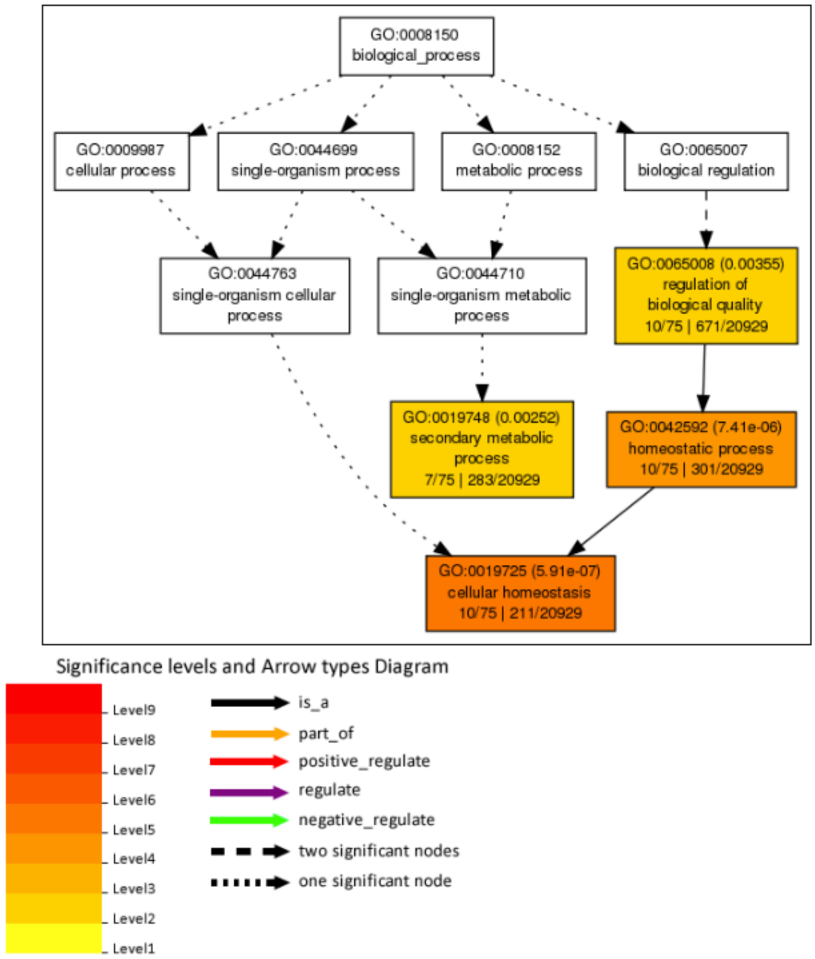

Figure S5. Results of Singular Enrichment Analysis (SEA) for the 75 out of 104 down-regulated transcripts between 1103P and SO4 grown in absence of Mg (-Mg) for 4 days with GO terms; the analysis was performed using agriGO v2.0 [126] using the locus ID (Gramene Release 5.0) as reference, Fisher as statistical test method, Yekutieli (FDR under dependency) as Multi-test adjustment method, 0.05 as significant level, 5 as minimum number of mapping entries and Plant GO slim as Gene Ontology type.

Figure S6

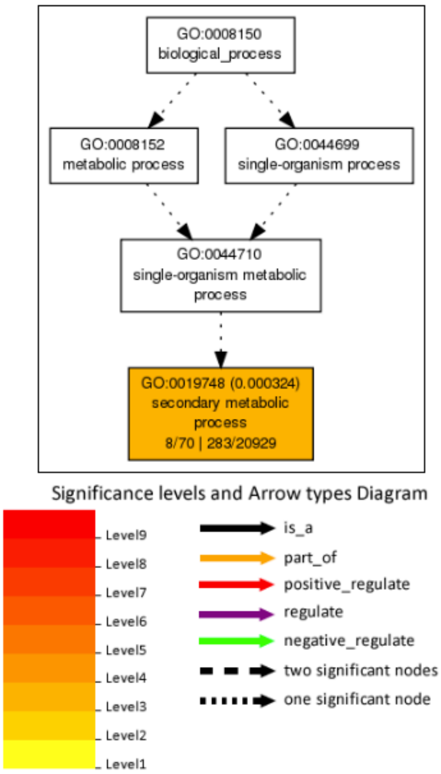

Figure S6. Results of Singular Enrichment Analysis (SEA) for the 70 out of 128 up-regulated transcripts between 1103P and SO4 grown in absence of Mg (-Mg) for 14 days with GO terms; the analysis was performed using agriGO v2.0 [126] using the locus ID (Gramene Release 5.0) as reference, Fisher as statistical test method, Yekutieli (FDR under dependency) as Multi-test adjustment method, 0.05 as significant level, 5 as minimum number of mapping entries and Plant GO slim as Gene Ontology type.

Figure S7

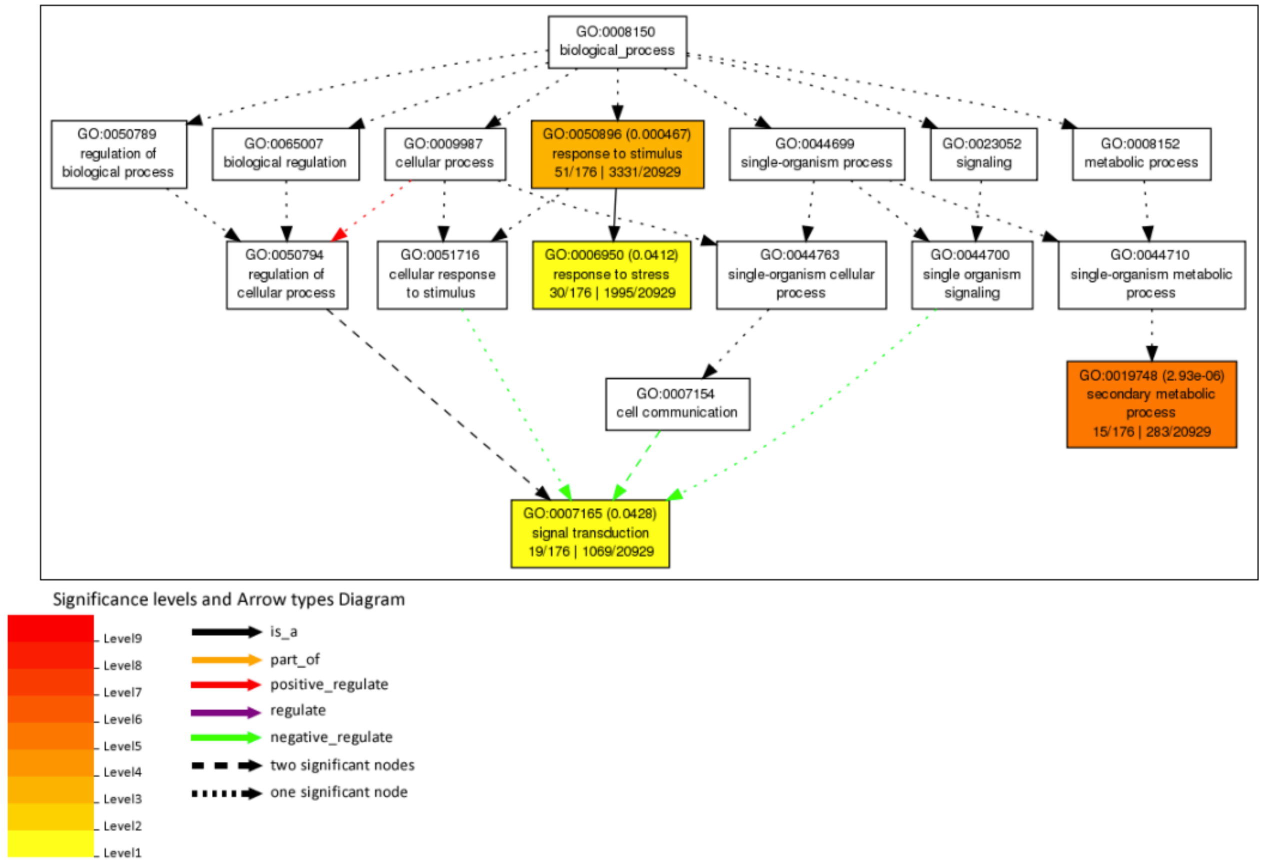

Figure S7. Results of Singular Enrichment Analysis (SEA) for the 176 out of 233 down-regulated transcripts between 1103P and SO4 grown in absence of Mg (-Mg) for 14 days with GO terms; the analysis was performed using agriGO v2.0 [126] using the locus ID (Gramene Release 5.0) as reference, Fisher as statistical test method, Yekutieli (FDR under dependency) as Multi-test adjustment method, 0.05 as significant level, 5 as minimum number of mapping entries and Plant GO slim as Gene Ontology type.

Figure S8

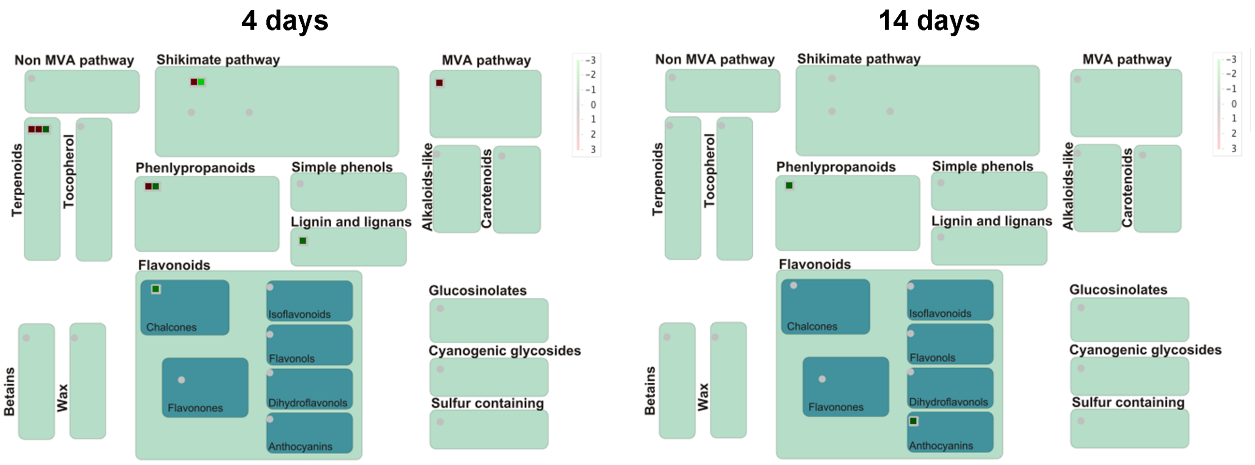

Figure S8. MapMan overview display of differentially expressed transcripts between 1103P and SO4 concerning the secondary metabolism. The Log_2(ratio) is shown by the color scale (green indicates a decrease and red an increase in transcript abundance between 1103P and SO4). The analysis was carried out using MapMan software [123].
