# Supplementary material for: The different tolerance to magnesium deficiency of two grapevine rootstocks relies on the ability to cope with oxidative stress
Source: BMC Plant Biol. 2019 Apr 16;19:148. doi: 10.1186/s12870-019-1726-x (PMC6469136; doi:10.1186/s12870-019-1726-x)
Supplement: Supplementary file 9 — Table S8. Real-time RT-PCR of a set of transcripts differentially expressed between roots of 1103P and SO4 at 4 and 14 days of growth in presence (+Mg) and absence (-Mg) resulted differentially expressed by microarray analysis. Probe ID, V1 transcript ID, description, microarray fold change (FC) value and RT-PCR data (means ± SE of three biological replicates) were reported. Table S9. Sequence of forward and reverse primers used in Real-time RT-PCR experiments. Probe ID, V1 transcript ID, description, 5’-3’ nucleotide sequence of forward and reverse primers were reported. (PDF 1310 kb) [file 12870_2019_1726_MOESM9_ESM.pdf]

Table S8

1103P +Mg *vs* SO4 +Mg; 4 days				
**Probe ID**	**V1 transcript ID**	**Description**	**Microarray FC**	**Real-time RT-PCR FC**
CHR3_JGVV38_152_T01	VIT_03s0038g03180	Flavin-containing monooxygenase	-2.01	-4.15 ± 0.59
**1103P -Mg *vs* SO4 -Mg; 4 days**				
**Probe ID**	**V1 transcript ID**	**Description**	**Microarray FC**	**Real-time RT-PCR FC**
CHR11_JGVV103_64_T01	VIT_11s0103g00010	Sodium transporter family protein	2.35	1.38 ± 0.40
CHR14_JGVV66_153_T01	VIT_14s0066g01670	Putative alpha-dioxygenase family protein	-2.06	-1.76 ± 0.01
CHR18_JGVV1_805_T01	VIT_18s0001g11430	Cytochrome P450 CYP82D47	-3.90	-5.41 ± 1.01
**1103P +Mg *vs* SO4 +Mg; 14 days**				
**Probe ID**	**V1 transcript ID**	**Description**	**Microarray FC**	**Real-time RT-PCR FC**
CHR7_JGVV31_256_T01	VIT_07s0031g02820	Magnesium transporter CorA	2.04	18.22 ± 1.42
**1103P -Mg *vs* SO4 -Mg; 14 days**				
**Probe ID**	**V1 transcript ID**	**Description**	**Microarray FC**	**Real-time RT-PCR FC**
CHR13_JGVV106_43_T01	VIT_13s0106g00580	Potassium channel AKT1	-2.91	-2.59 ± 0.11
CHR13_JGVV67_99_T01	VIT_13s0067g02360	Peroxidase	-2.55	-5.48± 1.71
CHR19_JGVV93_24_T01	VIT_19s0093g00350	Glutathione S-transferase	2.13	2.52 ± 0.26

Table S9

Probe ID	V1 transcript ID	Description	Forward primer	Reverse primer
CHR11_JGVV103_64_T01	VIT_11s0103g00010	Potassium-sodium symporter HKT2	5'-AGACCCCCTAAACTTCAGTG-3'	5'-CTCCATCTTCCAACAAATCCA-3'
CHR13_JGVV106_43_T01	VIT_13s0106g00580	Potassium channel AKT1	5'-TGGAAGGAGTTTTGGTGGAG-3'	5'-TTGCTGTCGGATTCATTTGG-3'
CHR13_JGVV67_99_T01	VIT_13s0067g02360	Peroxidase	5'-TGGAGAGATTAGGAAGAACTG-3'	5'-CAAGGATGGTGATACAAAGGG-3'
CHR14_JGVV66_153_T01	VIT_14s0066g01670	Putative alpha-dioxygenase family protein	5'-TTCATTGACTGAGGAGTTTAC-3'	5'-AAGGGTGGAGATTTGTTGAG-3'
CHR18_JGVV1_805_T01	VIT_18s0001g11430	Cytochrome P450 CYP82D47	5'-TTGATATGAGCGAGAGTTTTGG-3'	5'-CAAGCGTGGGGAGATAAGAA-3'
CHR19_JGVV93_24_T01	VIT_19s0093g00350	Glutathione S-transferase	5'-CCCTACTTTGTCTCTCTGTA-3'	5'-CTTGTCGTTTTGCTCCCCTT-3'
CHR3_JGVV38_152_T01	VIT_03s0038g03180	Flavin-containing monooxygenase	5'-CTACTGCCCATCCTAAAATCA-3'	5'-ACCTCTTTCCATTCTTCCTTTC-3'
CHR7_JGVV31_256_T01	VIT_07s0031g02820	Magnesium transporter MRS2-F	5'-AAGAAGAAAAAACTGTTGGGTT-3'	5'-TATAAAGCATAAAAGACAAGGCG-3'
	VIT_08s0040g00980	Tubulin beta chain	5'-CCCCTCTTCCTTTACTATGA-3'	5'-TCTGGCACTATTACAACTGG-3'
	VIT_06s0004g03220	Elongation factor 1-alpha	5'-GTGCGTCATAGTTTTCTGCC-3'	5'-AAAGAGGACACGACACAACAT-3'
